# Preparation of pyrite concentrate powder from the Thackaringa mine for quantitative phase analysis using X-ray diffraction

**DOI:** 10.1107/S1600576722009888

**Published:** 2022-11-29

**Authors:** Hamish McDougall, Monica Hibberd, Andrew Tong, Suzanne Neville, Vanessa Peterson, Christophe Didier

**Affiliations:** a School of Chemistry, The University of New South Wales, Anzac Parade, Sydney, NSW 2052, Australia; b Australian Centre for Neutron Scattering, Australian Nuclear Science and Technology Organisation, New Illawarra Road, Sydney, NSW 2232, Australia; c Cobalt Blue, 17.03 100 Miller Street, North Sydney, NSW 2060, Australia; Australian Synchrotron, ANSTO, Australia

**Keywords:** X-ray diffraction, quantitative phase analysis, pyrite mineral, sample preparation

## Abstract

The quantitative phase analysis using X-ray diffraction of pyrite ore concentrate samples extracted from the Thackaringa mine is problematic due to poor particle statistics, microabsorption and preferred orientation. The influence of sample preparation on these issues has been evaluated, with ball milling of the powder found most suitable for accurate and precise quantitative phase analysis. The milling duration and other aspects of sample preparation have been explored, resulting in accurate phase reflection intensities when particle sizes are below 5 µm. Quantitative phase analysis on those samples yielded precise phase fractions with standard deviations below 0.3 wt%. Some discrepancy between the elemental composition obtained using X-ray powder diffraction data and that determined using wavelength-dispersive X-ray fluorescence was found, and is thought to arise from unaccounted for crystalline phase substitution and the possible presence of an amorphous phase. This study provides a methodology for the precise and accurate quantitative phase analysis of X-ray powder diffraction data of pyrite ore concentrate from the Thackaringa mine and a discussion of the limitations of the method. The optimization process reveals the importance of confirming reproducibility on new samples, with as much prior knowledge as possible.

## Abstract 2

A method for performing accurate quantitative phase analysis using X-ray powder diffraction of pyrite concentrate mineral samples from the Thackaringa mine has been devised. The sample preparation optimization process is described. Grain size reduction suitable for analysis by the Rietveld method was achieved using ball milling.

## 1. Introduction

With recent shifts towards mining of lower grade ore deposits (West, 2011 ▸; Summerfield, 2020 ▸), mineral characterization that is both precise and accurate is becoming increasingly valuable. Deteriorating ore quality has led to more complex multiphase mineral compositions, which require representative crystalline phase information for the design of effective strategies for the post-processing and elemental recovery of newly tapped sources. X-ray diffraction (XRD) is one of the most common techniques used for the characterization of mineral samples, being simple and relatively fast.

Quantitative phase analysis (QPA) from whole-pattern fitting using the Rietveld method (Rietveld, 1969 ▸; Werner *et al.*, 1979 ▸) is routinely used to extract information such as phase identification, weight fractions and unit-cell parameters of crystalline phases within a powder sample. The accuracy and reliability of QPA results depend strongly on sample preparation (Dermatas *et al.*, 2007 ▸; Madsen *et al.*, 2019 ▸). One important requirement is that the irradiated sample volume contains a sufficient number of randomly oriented crystallites. This may be prevented by poor particle statistics, preferred orientation and microabsorption, well known factors discussed in the literature (Klug & Alexander, 1954 ▸; Bish & Reynolds, 1989 ▸; Moore & Reynolds, 1997 ▸; Zhang *et al.*, 2003 ▸; Dermatas *et al.*, 2007 ▸; Kleeberg *et al.*, 2008 ▸; Madsen *et al.*, 2019 ▸). A number of preparation techniques have been developed to resolve those issues in multiphase samples (Klug & Alexander, 1954 ▸; Bish & Reynolds, 1989 ▸; Moore & Reynolds, 1997 ▸; Hillier, 1999 ▸; Monecke *et al.*, 2001 ▸; Dermatas *et al.*, 2007 ▸; Kleeberg *et al.*, 2008 ▸).

Because the optimal method of sample preparation depends on sample characteristics such as composition, phase distribution, particle size and morphology, the preparation is often devised by trial and error and has not been considered for pyrite ore concentrates. In this work we evaluate and optimize the sample preparation for powder XRD of pyrite ore concentrate from the Thackaringa mine, Broken Hill (NSW, Australia), and estimate absolute and relative uncertainties on phase weight fractions extracted by QPA.

## 2. Materials and methods

### 2.1. Sample preparation

The pyrite concentrate used in this study was produced from ore originating from the Thackaringa mine in Broken Hill, New South Wales, Australia, taken from a pyrite–quartz–albite gneiss at the ‘Pyrite Hill’ site in February 2019, containing approximately 20% pyrite by weight. The raw ore obtained by reverse cycle drilling was crushed and treated by gravity separation, followed by a scavenger float on the gravity tails. The gravity and float concentrates were combined to form the pyrite concentrate sample. The resulting coarse powder was stored under water at room temperature prior to handling.

Approximately 1 kg aliquots of this powder were washed with deionized water, vacuum filtered and dried at approximately 353 K for 3–5 h, then stored in closed containers at 258 K. Ore content variation is artificially introduced between aliquots due to manual sampling. To minimize this ore content variation, all measured samples were taken from the same aliquot roughly homogenized by rotation of the container. Samples were hand ground using an agate mortar and pestle for 25 min. Samples were ball milled using a tungsten carbide (WC) jar and balls in an ESSA LM1-P vibratory ball mill, where 15 g of material was milled for 1, 2, 3, 5, 7 or 10 min. Magnetic separation of antiferromagnetic pyrrhotite from pyrite ore concentrate was performed by vigorously shaking 10 g sample aliquots in an inverted sample vial with a neodymium magnet on top of the vial.

For comparison purposes, 15 g of pyrite ore concentrate was milled using a jar of alumina rods in a McCrone micronizing mill in 7 ml of propan-2-ol for 30 min. The sample was rinsed from the container with additional propan-2-ol (5–10 ml), which was then evaporated by heating to 353 K for 1 h.

### 2.2. XRD measurement

XRD measurements were performed using a Malvern Panalytical Empyrean II diffractometer with a Co *K*α X-ray source (λ = 1.7891 Å) and point detector in Bragg–Brentano geometry in the 10–120° 2θ range with the sample rotated at 30 rev min^−1^ during measurement. Apart from the grease-prepared samples described below, all powders were partly top loaded into 30 mm diameter × 1 mm deep stainless steel holders, covered with a glass slide and turned vertical with slight tapping to reorient the grains. Loading and vertical taps were repeated until the holder was full, after which the glass slide was removed from the horizontal holder and a razor blade taken across the surface. The grease-prepared samples used silicone high-vacuum grease (Chem Supply) to coat the base of a ‘zero background’ oriented silicon sample holder (Malvern Panalytical) onto which the sample was sprinkled in line with the lip of the holder, before gentle tapping to deposit a relatively thin layer of sample onto the grease.

The proportion of amorphous content was estimated by the external standard method (O’Connor & Raven, 1988 ▸; Scarlett & Madsen, 2018 ▸). Crystalline α-Al_2_O_3_ (Baikalox, CR1, Baikowski) powder was loaded into sample holders and measured identically to the pyrite concentrate samples. The crystallinity of the alumina standard was assumed to be 99.0 (6)% according to previous amorphous content determination of this material (Cline *et al.*, 2011 ▸). The overall profile scale factor was obtained by the Rietveld method as described in Section 2.3 using the ICDD (International Centre for Diffraction Data, Newtown Square, Pennsylvania, USA; http://www.icdd.com) 01-070-7019 (Pillet *et al.*, 2001 ▸) crystal structure for α-Al_2_O_3_; refined unit-cell and atomic parameters for this phase are given in Table S5 in the supporting information. The mass attenuation coefficients of the standard and concentrate were calculated according to their elemental composition using the database of Chantler (2001 ▸).

### 2.3. XRD analysis

Crystalline phases in the XRD data of the pyrite concentrate powder were identified using Malvern Panalytical’s *Highscore* semi-quantitative analysis software with the ICDD PDF4+2021 database and the ICSD [Inorganic Crystal Structure Database, FIZ-Karlsruhe, Germany, and the National Institute of Standards and Technology (NIST), USA; https://icsd.fiz-karlsruhe.de/index.xhtml] release 2020.1. QPA was performed using the Rietveld method (Rietveld, 1969 ▸) as implemented in the *GSAS-II* software (Toby & Von Dreele, 2013 ▸). The instrumental peak shape was modelled using a pseudo-Voigt function with *W*, *X*, *Y*, instrumental zero and asymmetry parameters determined from data for a silicon standard (Malvern Panalytical) and fixed in all subsequent refinements of sample data. The background was modelled with a seven-coefficient Chebyshev 1 polynomial and the vertical sample displacement refined.

The crystal structures used as starting structures in the phase refinements were ICDD 04-014-3191 (Wu *et al.*, 2004 ▸) for pyrite (FeS_2_), ICSD 162490 (Antao *et al.*, 2008 ▸) for quartz (SiO_2_), ICSD 68913 (Armbruster *et al.*, 1990 ▸) for albite (NaAlSi_3_O_8_), ICDD 00-029-0723 (Morimoto *et al.*, 1975 ▸) for pyrrhotite (Fe_7_S_8_) and ICSD 64987 (Shintani *et al.*, 1975 ▸) for rutile (TiO_2_). Each phase had its unit cell, crystallite size and phase fraction refined. Refinement of the triclinic unit cell of albite proved unstable, so the unit-cell parameters of albite were not refined for samples milled for 0, 1, 2 and 3 min but could be refined at the last refinement step for the 5, 7 and 10 min milled samples. A March–Dollase parameter was included in the refinements to correct for the severe preferred orientation observed for albite, with the direction [013] yielding better agreement between calculated and observed patterns, as consistent with previous analysis (Kleeberg *et al.*, 2008 ▸). For rutile, given its low phase fraction (<1 wt%), it was necessary to fix the crystallite size to an appropriate value (0.08 µm) for refinement of the samples milled for 0 min to avoid refinement divergence, but the parameter could be refined for samples milled for 1, 2, 3, 5, 7 and 10 min. Refinement of coordinates and isotropic atomic displacement parameters (ADPs) for quartz, albite, pyrrhotite and rutile often resulted in refinement instability or non-sensible chemical composition, so these were not refined. Atomic coordinates and ADPs for the pyrite phase in the grease-prepared samples and those samples milled for 5, 7 and 10 min could be refined, with negligible change to phase fraction and crystallite size values when those were refined.

### 2.4. Neutron diffraction (ND)

ND measurements were performed on the Wombat high-intensity neutron powder diffractometer (Studer *et al.*, 2006 ▸) at the Australian Nuclear Science and Technology Organisation’s Centre for Neutron Scattering, Lucas Heights (NSW, Australia). Powder samples were loaded into 9 mm diameter vanadium cans and suspended in the neutron beam of wavelength 1.5430 (1) or 2.4144 (3) Å determined using the La^11^B_6_ Standard Reference Material (SRM) 660b from NIST. Rietveld refinements using neutron diffraction data have been attempted, but the precise determination of phase weight fractions in those data was prevented by difficulty in observing reflections from minor phases as a result of un­favourable neutron scattering cross sections and instrumental broadening.

### 2.5. Wavelength-dispersive X-ray fluorescence (WD-XRF)

WD-XRF measurements were performed using a Malvern Panalytical Axios Advanced WDXRF instrument with a Rh *K*α X-ray source (λ = 0.615 Å). Samples of 10–12 g were prepared by milling for 7 min as per Section 2.1. A 10:1 mass ratio of sample to Ceridust 3620 (polyethylene wax, used as a binding agent) was mixed and pressed into a pellet, and a 1074.7 mm^2^ area was illuminated during measurement. Two types of WD-XRF analyses were made: semiquantitative multi-elemental analysis, and quantitative analysis calibrated for Fe, S and Si.

The multi-elemental analysis provides semiquantitative elemental ratios for elements between Be and U using a fundamental parameters matrix correction (Omnian) calibrated in-house by Malvern Panalytical. In this analysis, lighter elements are slightly overestimated, probably as a result of the stronger X-ray attenuation of the pyrite concentrate compared with that of the standard (Rousseau, 2006 ▸), noting that ratios between elements with close *Z* are less affected. The automatic peak search and match were corrected by visual inspection of the spectra. Peaks for rhodium and phosphorus, arising from the source X-ray tube and Ceridust binder, respectively, were excluded from the analysis.

The quantitative calibrated analysis measured the Fe, S and Si content using empirical calibration curves from mixtures that matched the composition and sample preparation method of the concentrate, comprising three standards of 70:30, 80:20 and 90:10 ratios of FeS_2_:SiO_2_. These standards were produced using pyrite (iron disulfide, 99.8%, Sigma Aldrich) and acid-washed quartz (laboratory grade, Chem Supply), where 10 g of coarse quartz was first milled for 1 min and ground together with the appropriate mass of pyrite in an agate mortar before shaking in a glass vial. Pellets of this mixture were then milled and pressed identically to the pyrite concentrate samples for WD-XRF measurements. Calibration curves were obtained by linear regression between measured *K*α peak intensities for Fe, S and Si and the corresponding weighed concentrations of standard mixtures.

### 2.6. Scanning electron microscopy and energy-dispersive spectroscopy (SEM-EDS)

For particle surface imaging, samples were sprinkled onto a two-sided tape-covered mount. For particle cross-section imaging and elemental mapping, powder samples were covered in epoxy resin precursors, evacuated for 10 min and left to cure overnight. Particles were exposed by abrading the surface of cured resin samples for 1 to 5 min using P800 sandpaper; the samples were then cleaned under sonication in a distilled water bath, polished for 5 min using P1200 sandpaper and cleaned in the same manner once more. Dried resin samples were mounted using aluminium tape and coated with 10 nm of carbon using a Safematic CCU-010 compact coating unit immediately prior to analysis. SEM imaging was performed with an ST Instruments TM4000Plus tabletop microscope using a backscattered electron (BSE) detector paired with a Bruker QUANTAX energy-dispersive X-ray spectrometry (EDS) detector. Ten SEM images were taken of each pyrite concentrate powder ball milled for different times.

### 2.7. Inductively coupled plasma mass spectrometry (ICP-MS)

Approximately 1 g of sample was microwave digested in aqua regia mixture (3HCl + 2HNO_3_) to ensure all soluble materials were dissolved. It was noted that the sample contained some insoluble particles, most likely silicates. ICP-MS measurements were performed on the extracted solution using a Perkin Elmer NexIon 300D instrument.

### 2.8. Particle size analysis

Particle size distribution analysis was performed using a Malvern Panalytical Mastersizer 3000 laser dispersion instrument. Approximately 5 × 10^−4^ g ml^−1^ of untreated pyrite ore concentrate was used for dispersion in deionized water. Aliquots of sample were added slowly until the instrument reached the required particle obscuration rate (the rate at which specified surface can be accurately calculated and the risk from deposited particles is reduced). The samples were automatically sonicated and dispersed by the instrument during measurements, with blank measurements taken between each individual sample. Measurements were replicated three times for each sample to determine experimental uncertainty.

### 2.9. Sieve analysis

Sieve analysis was performed on unground pyrite concentrate using a series of Cole–Parmer stainless steel standard test sieves (ASTM E11 approved) with mesh sizes of 75, 106, 250, 600 and 1000 µm, using a Laarman LMSM 75–240 V/50 Hz horizontal sieve shaker for 1 h. The particle size distribution was obtained by weighing the material collected in each sieve [Fig. S2(*a*)]. Phase fractions in each portion were obtained from QPA using XRD following the optimized sample preparation outlined above.

## 3. Results and discussion

### 3.1. Qualitative analysis of samples

**Figure 1:**
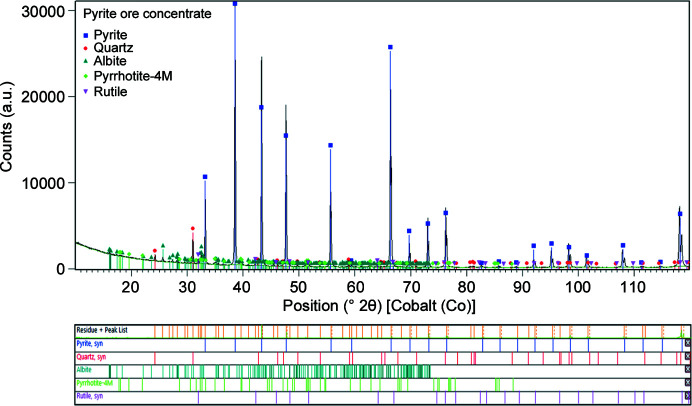
Phase identification using the *Highscore* software analysis of XRD data of pyrite concentrate powder ball milled for 7 min. Data are shown as a black line and all peaks can be indexed to one of five crystalline phases.

Before QPA was performed, the crystalline phases present in the sample needed to be identified. A powder diffraction pattern was initially obtained from XRD using a sample prepared by hand grinding in an agate mortar and top loading into a flat horizontal sample holder. This preparation was later found inappropriate for accurate QPA, as shown in Section 3.2, with ball milling necessary to obtain phase reflection intensities closer to those expected from published crystal structures. This gradual process was necessary to devise an optimal measurement strategy, with knowledge of the sample mineral content and particle distribution informing the preparation methods. XRD data for the pyrite concentrate sample ball milled for 7 min were used to identify crystalline phases present (Table 1 ▸ and Fig. 1 ▸), with elemental compositions corroborated with XRF and SEM-EDS.

Five crystalline phases were identified from the XRD data, with all reflections indexed by this phase composition. Qualitatively, pyrite was found to be the major phase, with albite and quartz as minor phases. Pyrrhotite and rutile were present as trace phases. Observed reflection intensities for albite were closest to those expected for the low albite polymorph (Armbruster *et al.*, 1990 ▸). The 4M polymorph of pyrrhotite (Morimoto *et al.*, 1975 ▸) was identified from the XRD pattern of a magnetically separated sample (Fig. S1).

**Figure 2:**
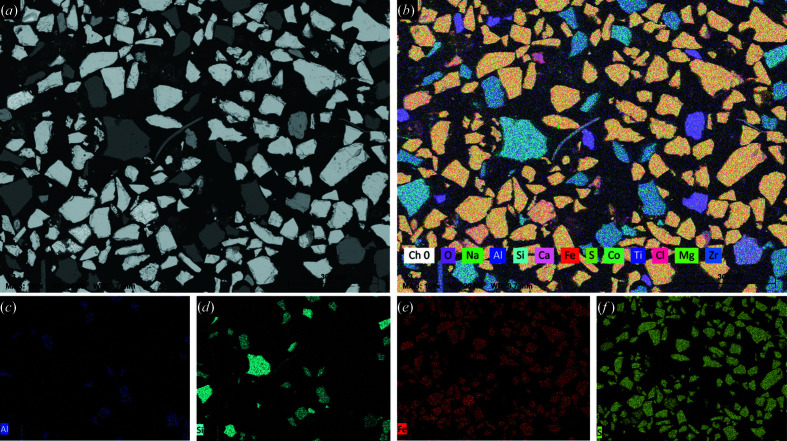
(*a*) SEM BSE image and (*b*) the corresponding elemental mapping (EDS) of unground pyrite concentrate powder encased in resin. (*c*)–(*f*) Individual maps for Al, Si, Fe and S, respectively. Ten areas and the corresponding elemental maps were measured and are shown in Fig. S3, and this area was chosen as representative.

The elemental composition of the pyrite concentrate obtained from semi-quantitative WD-XRF (for samples milled for 7 min, Table 2 ▸) and SEM-EDS (for as-received unground samples) was used to corroborate the phase identification obtained using the XRD data. Several representative images of the sample and the corresponding elemental compositions are shown in Fig. 2 ▸.

SEM imaging reveals phases separated into distinct particles of a relatively broad size distribution ranging from 1000 µm down to 1 µm. The measured composition of individual grains obtained using SEM-EDS is in agreement with the phase identification from XRD. The majority contain exclusively Fe and S, corresponding to the pyrite and pyrrhotite phases. The second most common compositions were silicates containing either Si and O, or Na, Al, Si and O, corresponding to the quartz and albite phases, respectively. A small number of aluminosilicate particles containing K or Ca (Fig. S3) suggests the presence of alkaline and plagioclase feldspars, noting significant miscibility between those minerals and albite, forming the feldspar family (Iddings, 1898 ▸; Zambonini & Washington, 1923 ▸). Other aluminosilicate particles con­taining Mg with lamellar morphology (Fig. S3) may suggest the presence of magnesium-rich mica, as observed in the ore before flotation (Plimer, 1977 ▸). No reflections for alumino­silicates other than albite were visible in the diffraction patterns as a result of their low amount, later confirmed using WD-XRF. SEM-EDS reveals Ti and O in isolated particles (light green in Fig. S3), confirming the presence of the rutile phase. Co is known to substitute for Fe in pyrite, and although no Co was detected in particles other than the iron sulfides, SEM-EDS has little discrimination between Fe and Co due to the overlap of Fe *K*β (7058 eV) and Co *K*α (6923 eV) (Deslattes *et al.*, 2005 ▸) transition energies. A few isolated oxide particles containing Zr, or a mixture of La, Ce and Nd, were sometimes observed using BSE imaging. However, no Zr or lanthanoid oxides were visible using XRD, presumably because of their very low amount as confirmed by WD-XRF (Table 2 ▸).

All elements >0.5 wt% as determined using WD-XRF (S, Fe, Si, Al, Na, Ti) correspond to phase compositions detected using XRD. Other elements (Co, Ni, Ca, Mg, K, Zr, Nb, Y) consistent with those detected by SEM-EDS were identified by WD-XRF but did not correspond to the composition of crystalline phases identified using XRD, presumably because these elements are present as substituents within visible phases or belong to impurity phases below the detection limit of the method. The higher energy resolution of WD-XRF compared with SEM-EDS enabled the confirmation of Co in the presence of Fe in the sample and also revealed trace amounts of Ni, possibly as a third substituent for Fe in the pyrite phase, in the molar ratio Fe:Co:Ni = 0.987 (8): 0.012 (10):0.002 (4). Trace amounts of Ca, K and Mg, alongside Na and Al, in the molar ratio Al:Na:Ca:K:Mg = 1.00 (2):0.91 (5):0.030 (9):0.02 (7):0.037 (5) are consistent with the presence of albite and the absence of other feldspars in the XRD data, and consistent with previous mineralogical analysis of samples from the Thackaringa mine (Plimer, 1977 ▸). The presence of trace amounts of Zr and Nb confirms the absence of visible zirconia and lanthanoid oxide phases in the XRD data. Trace amounts of As, Pb and Y may be present, but the signal is at the detection limit of the method (Fig. S4). As can substitute for S in FeS_2_, Pb may be a small impurity as PbS, commonly associated with pyrite (Abraitis *et al.*, 2004 ▸), and Y is a common impurity in lanthanoid minerals. We note the presence of W, probably as contamination from the WC jars used in the milling process.

### 3.2. Influence of preparation methods on quantitative phase analysis from XRD

**Figure 3:**
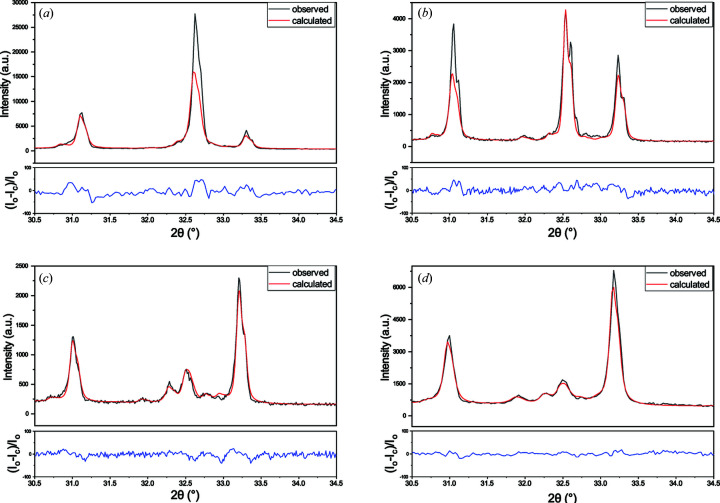
Rietveld refinement profiles using XRD data of pyrite concentrate powder as (*a*) an unground control, and prepared by (*b*) hand grinding, (*c*) grease loading and (*d*) 7 min ball milling. The relative percentage differences between the observed (*I*_o_) and calculated (*I*_c_) intensities are shown beneath each profile. Rietveld refinement residuals for each sample preparation are given in Table S2.

For comparison with milled samples, we first prepared pyrite concentrate samples for XRD by hand grinding powder for 30 min in an agate mortar and top loading into sample holders. As expected, XRD data for hand-ground preparations lacked reproducibility as a result of poor particle statistics, microabsorption and preferred orientation (Fig. S5). Reflection intensities differed slightly from those calculated for published crystal structures of pyrite and quartz, and differed greatly for albite (Fig. 3 ▸). Albite is known to be susceptible to preferred orientation due to its plate-like morphology and polysynthetic twinning (Heidelbach *et al.*, 2000 ▸; Jiang *et al.*, 2000 ▸; Kleeberg *et al.*, 2008 ▸; Vance, 1961 ▸; Donnay, 1940 ▸), with preferred orientation strong along the [001] direction and relatively weaker along the [010] direction in flat-plate sample preparations (Kleeberg *et al.*, 2008 ▸). Although preferred orientation in albite may be better represented by complex models such as spherical harmonics, considering reflections for this phase have low intensity, a single March–Dollase parameter was selected as an approximation to reduce the number of refined parameters. The best agreement between measured and calculated reflection intensities was obtained with a correction along the [013] unique axis by trial and error for the 0 and 1 min samples, roughly approximating the preferred orientation determined by Kleeberg and co-workers, noting that a range of [0*kl*] unique axes when *l* > *k* adequately modelled the observed intensities. The choice of unique axis becomes insignificant as milling time increases and preferred orientation becomes negligible (the March–Dollase parameter is close to 1). For consistency, refinement of preferred orientation along the [013] unique axis was used in model descriptions of all sample data.

Several methods for the preparation of samples for XRD analysis have been suggested to correct for this effect, such as side loading of samples or sprinkling onto grease (Kleeberg *et al.*, 2008 ▸; Unruh & Forbes, 2019 ▸). We note that top and side loading amplified different sets of reflections for albite, with the cluster of reflections at 32.6° the most susceptible, but neither method prevented preferred orientation. Loading onto grease substantially reduced preferred orientation in the albite phase (Fig. 3 ▸), where the March–Dollase parameter refined to a value closer to 1, indicating a more random orientation of the powder. However, the patterns still lacked reproducibility, with this more evident for pyrite reflections, suggesting that the relatively small amount of powder in the grease preparation and the large particle size may lead to poor particle statistics. Fig. 3 ▸ shows typical Rietveld refinement profiles using XRD data of pyrite concentrate powder prepared by four different methods. Refinement residuals and the March–Dollase correction value for albite are given in Table S2.

Although pyrite is relatively soft (around 6 on the Mohs scale) (Craig & Vokes, 1993 ▸), it was later confirmed by SEM and laser diffraction that the particles remained relatively large after hand grinding in an agate mortar, perhaps due to the presence of harder quartz particles (around 7 on the Mohs scale) (Deer *et al.*, 1962 ▸). Large crystallites are a well known source of systematic errors in powder diffraction (Moore & Reynolds, 1997 ▸). Neutron diffraction measurements with area detectors confirmed the poor particle statistics, even in larger pyrite concentrate samples, compared with XRD (Fig. S6). Although sieving is sometimes suggested to remove large crystallites (Brindley, 1945 ▸), pyrite concentrate powders are found to have a phase distribution that is correlated with grain size [Fig. S2(*b*)], with pyrite more represented in larger grains, so sieving would result in a modification of the sample composition. Grain size reduction has been shown to be effective in improving particle statistics and reducing preferred orientation and microabsorption (Madsen *et al.*, 2019 ▸; Kleeberg *et al.*, 2008 ▸; Klug & Alexander, 1954 ▸). A homogeneous size distribution of fine particles (<10 µm) has been stated as ideal (Brindley, 1945 ▸; Klug & Alexander, 1954 ▸). However, excessive milling can lead to the introduction of particle strain or induce chemical reactions, such as oxidation of ultrafine particles (Karim *et al.*, 2016 ▸; Dettrick *et al.*, 2019 ▸), and can also introduce structural defects, potentially causing further difficulty in QPA using XRD (Sakher *et al.*, 2018 ▸; Madsen *et al.*, 2019 ▸; O’Connor & Chang, 1986 ▸; Hillier, 2003 ▸; Dermatas *et al.*, 2007 ▸).

Better agreement between observed and modelled reflection intensity, and improved pattern reproducibility across preparations, were obtained after ball milling the pyrite concentrate (Fig. 3 ▸), suggesting the suitability of this method of pyrite concentrate sample preparation for QPA using XRD. Because the optimal milling procedure is dependent on sample characteristics (O’Connor & Chang, 1986 ▸), the milling preparation was optimized for pyrite concentrate powder and the accuracy and precision of the QPA results were determined.

### 3.3. Optimization of sample milling time

**Figure 4:**
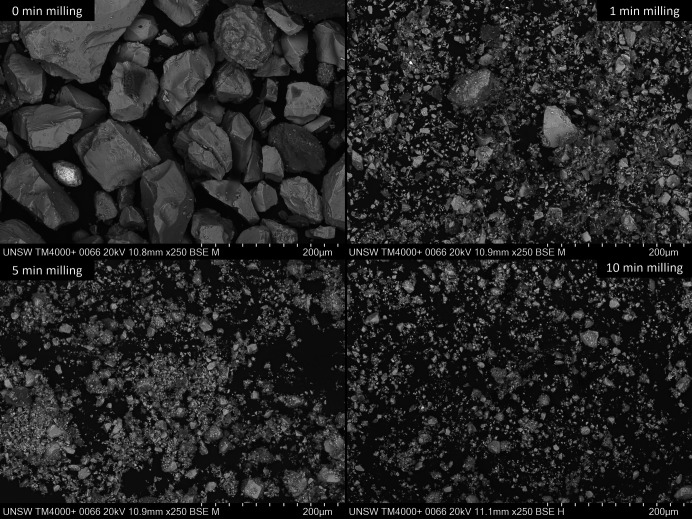
Representative SEM BSE images of pyrite concentrate powder samples ball milled for 0, 1, 5 and 10 min.

The particle size distribution of pyrite concentrate powder ball milled for different times was investigated by SEM imaging and laser diffraction (Fig. 4 ▸ and Table 3 ▸).

The SEM and laser diffraction data follow the expected trend of a reduction in particle size with increasing milling time, where the reduction rate decreases as the milling time is further increased (O’Connor & Chang, 1986 ▸). Powders milled for 5 and 10 min contain particles of <3 µm diameter in larger agglomerates, possibly due to triboelectric effects introduced by friction in the dry milling procedure (Mirkowska *et al.*, 2016 ▸; Landauer & Foerst, 2019 ▸), with a small portion of particles of diameter >10 µm. Laser diffraction reveals a multimodal particle size distribution (Fig. S18), probably resulting from differential grinding according to mineral hardness.

The pyrite concentrate powder milled for different times was examined using XRD, with the final phase composition calculated from Rietveld refinement using five separate XRD measurements of samples from the same batch of milled powder. Pattern simulations (Fig. S7) showed negligible changes in intensity when Co, Ni and As were substituted into the crystal structure of FeS_2_, or when Mg, Ca and K were substituted into NaAlSi_3_O_8_, according to semi-quantitative WD-XRF elemental ratios, suggesting that XRD is not sensitive to those substitutions. Given this lack of sensitivity, the refinement of atomic occupancies was not attempted and pure phase compositions were used for QPA.

**Figure 5:**
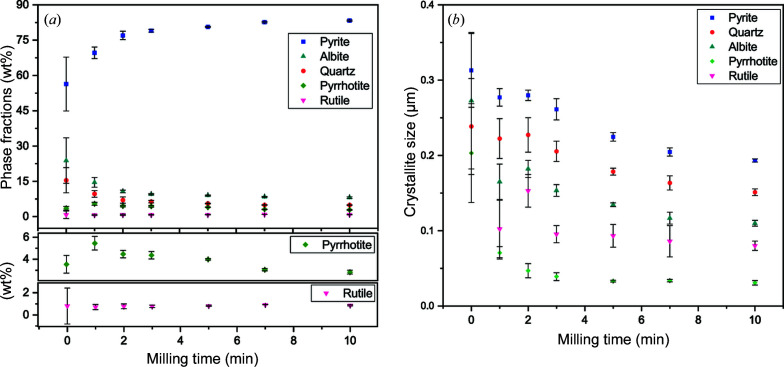
(*a*) Mean phase fractions and (*b*) mean crystallite sizes for phases determined using XRD of five pyrite concentrate powder samples milled for different times. For the 0 min milling time sample, the crystallite size of rutile was fixed to 0.1 µm to prevent correlations between crystallite size and weight fraction parameters. Error bars represent sample standard deviations of the mean values for the five repeats at each milling time. Values of the Rietveld refinement residual *R*_wp_ are plotted in Fig. S8 and given in Table S3 with other residuals. Typical refinement profiles are shown in Fig. S9–15.

**Figure 6:**
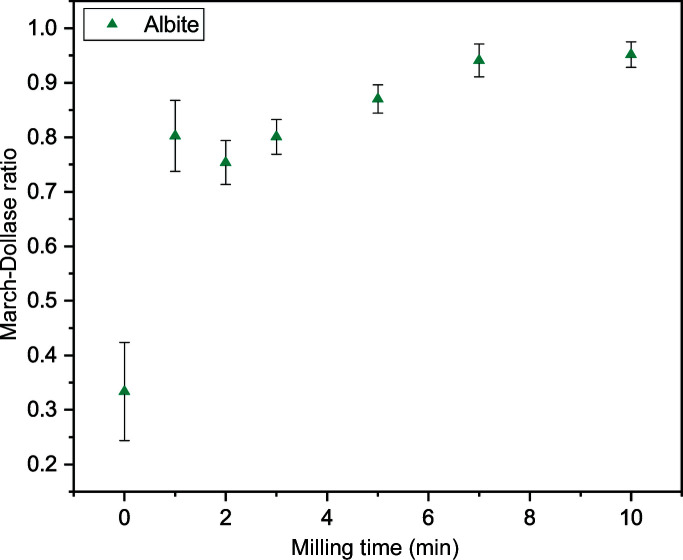
The mean March–Dollase ratio for the albite phase obtained from Rietveld refinements using XRD data of five pyrite concentrate powders milled for different times. A March–Dollase ratio of 1 indicates random powder orientation with respect to the chosen unique reflection direction [013]. Reflection intensities in very low milling time samples with poor particle statistics may also be partially modelled by this parameter. Error bars represent sample standard deviations of the mean values for the five repeats at each milling time. Values are given in Table S3.

The average results and reproducibility over the five repeat analyses are first qualitatively assessed before comparison with the composition obtained from calibrated WD-XRF. Standard deviations in refined parameters obtained for five replicates (Figs. 5 ▸ and 6 ▸) reveal a clear improvement in the reproducibility of XRD data when the sample milling time is increased. The discrepancy between observed and calculated patterns (Figs. S9–S15), as reflected in the weighted profile reliability factor *R*_wp_ (Fig. S8, Table S3), also decreases with increased milling time, with a plateau reached after about 7 min. Fig. 6 ▸ shows that the mean refined value of the March–Dollase parameter to correct for preferred orientation of the albite phase approaches 1, indicative of minimal preferred orientation in the phase, as the milling time increases. Excluding measurements with unground powder, the unit-cell parameter changes were within 0.002% (Table S4), suggesting chemical stability of the phases.

**Figure 7:**
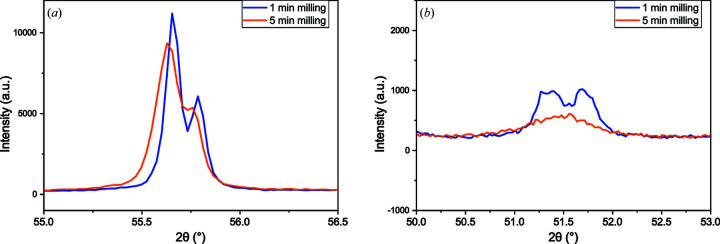
XRD data, shown as solid lines for clarity, of pyrite concentrate powder milled for 1 and 5 min, showing broadening of (*a*) the pyrite overlapped reflections 312 and 321, and (*b*) the pyrrhotite overlapped reflections 402, 223, 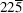 , 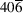 and 313.

Particle size reduction causes reflection broadening, as indicated by the reduction in the refined crystallite size with milling time [Fig. 7 ▸(*b*), Fig. S16]. Peak broadening may cause difficulty in discriminating reflections for minor phases such as rutile and pyrrhotite [Fig. 7 ▸(*a*)], with significant broadening for the pyrrhotite phase causing overlap of five proximal pyrrhotite reflections in the data [Fig. 7 ▸(*b*)] and refined crystallite sizes below 0.05 µm. This is probably due to pyrrhotite having a much lower hardness (around 3.5–4.5 on the Mohs scale) (Chen *et al.*, 2020 ▸) than the other phases. Reflection overlap from broadening may explain the increase in uncertainty observed for the pyrrhotite weight fraction when the milling time is increased from 7 to 10 min (Fig. 5 ▸). Milling times longer than 10 min were not investigated.

The preparation was compared with that obtained using the McCrone XRD-Mill vibratory rod mill under propan-2-ol, a particle size reduction method recommended for XRD sample preparation (Whitfield *et al.*, 2019 ▸). Mean particle sizes equivalent to those obtained after 7 min in the ESSA mill were obtained after 30 min in the McCrone mill (Fig. S18). Although laser diffraction revealed a narrower particle size distribution using the McCrone mill, under-ground particles of around 10 µm remained. Comparison of XRD data for samples ground using the two different mills revealed slightly sharper reflections for the McCrone preparation (Fig. S19), resulting in smaller refined crystallite sizes for most phases (Table S6). Refined parameters were comparable between the two preparations, although we note that further optimization of the faster ESSA ball mill method may be achieved by considering factors such as the number, size and shape of the balls, as well as the amount of powder, number of rotations per minute, milling medium, ball and jar materials *etc*.

A strong correlation exists between particle size and refined parameters (Fig. S16), as expected from the influence of particle statistics and microabsorption, noting a substantial underestimation of pyrite weight fraction when the particle size is high. Microabsorption (Brindley, 1945 ▸) is expected to be problematic in pyrite concentrate samples due to the presence of phases with different mass attenuation coefficients (MACs) (FeS_2_ MAC = 100 cm^2^ g^−1^, SiO_2_ MAC = 54 cm^2^ g^−1^ at 1.79 Å); we note that although corrections exist (Brindley, 1945 ▸; Rousseau, 2006 ▸) they are often unsatisfactory (Scarlett & Madsen, 2018 ▸). Although further particle size reduction may reduce microabsorption, it may also induce excessive broadening of reflections. Shorter-wavelength instruments may be desirable for those samples. A milling time of around 7 min, corresponding to mean particle sizes close to 3 µm, was considered a suitable compromise for the preparation of pyrite concentrate samples for XRD using Co *K*α wavelength.

### 3.4. Comparison of XRD results with elemental analysis

To confirm the validity of the refined phase fractions from XRD analysis, the results were compared with elemental analysis determined using WD-XRF. The precision of QPA from XRD was assessed by repeating the measurement and analysis over five samples taken from 1 kg aliquots of the pyrite concentrate that were subjected to 7 min milling and compared with WD-XRF analyses of the samples (Tables 4 ▸ and 5 ▸).

Table 4 ▸ shows the mean phase fractions obtained from QPA of five pyrite ore samples milled for 7 min. The standard deviations for the refined phase fractions over the five repeats lie below 0.3 wt%, confirming the good reproducibility of this preparation method. The elemental composition calculated from the refined phase fractions obtained from XRD is compared with that obtained using WD-XRF in Table 5 ▸. We note that calibration with a matrix-matching standard (Souders & Sylvester, 2010 ▸; Rousseau, 2006 ▸) was necessary in the WD-XRF method, with the major elements Fe, S and Si calibrated using FeS_2_/SiO_2_ mixtures in a composition range close to that of the pyrite concentrate sample. The elemental weight fractions for these elements obtained from the two methods are within 2 wt% (Table 5 ▸). Although in general agreement, the amount of Fe and S is slightly overestimated and Si slightly underestimated by XRD. The bias is much larger than the standard deviations across repeats (Table 5 ▸), suggesting a systematic, rather than statistical, error. Some of these discrepancies could be ascribed to elements not included in the composition of refined phases, such as Mg, K and Ca in albite, and Co, Ni and As in pyrite, or impurities containing Pb, Nd, Y and Zr in amounts too small to be visible in XRD. However, the difference should be smaller than 0.5 wt% according to multi-elemental WD-XRF data (Table 2 ▸). An overestimation of heavier elements may point to uncorrected microabsorption, despite empirical calibration being used in the XRF analysis, perhaps resulting from a different particle size distribution in the ore sample compared with purchased synthetic standards. Although particle size reduction decreases the effect of microabsorption, and samples were ball milled identically in preparations for both XRD and XRF analyses, microabsorption may still be non-negligible (Scarlett *et al.*, 2002 ▸; Whitfield, 2016 ▸) and the degree of microabsorption may differ between characterization methods considering the different wavelengths used.

The presence of an unaccounted for amorphous phase may also explain the differences between elemental compositions determined by XRD and WD-XRF, with this phase either present in the original material or induced by high-intensity ball milling. The external standard method (O’Connor & Raven, 1988 ▸) returned −9.0 (5) wt% amorphous content. This physically unrealistic negative value reveals a bias of the method, consistent with a previous investigation (Scarlett & Madsen, 2018 ▸) which showed that amorphous content estimation is notoriously inaccurate in complex samples susceptible to microabsorption, as is the case for pyrite ore concentrate samples. Correlations between refined ADPs and scale factors of the alumina standard [correlation factors were 0.81 and 0.66 for *U*_iso_(Al) and *U*_iso_(O), respectively] further contribute to inaccuracies (Madsen *et al.*, 2011 ▸).

Although the exact amount cannot be determined accurately, the absence of broad features in the background of the XRD patterns (Fig. S17) comparable to the fully crystalline standard suggests that the amount of amorphous phases is low in the concentrate. A bias can also be introduced from imperfectly modelled reflection intensity and peak shape. Crystal structures with calculated intensity ratios close to those observed were selected from the literature but some uncertainty remains, for example in the choice of polytype for albite being complicated by preferred orientation for this phase in pyrite concentrate samples. Both pyrite and albite are known to have a strong tendency to accept impurities, as suggested by multi-elemental XRF and SEM-EDS, with different compositional variances generating slightly different Bragg positions which, when superimposed, can result in slightly abnormal peak shapes that can hamper correct intensity refinement. Although refined parameters such as particle size or strain, March–Dollase correction, atomic positions and ADPs can model those differences, resulting in reasonable agreement between observed and calculated patterns, a systematic error may remain in refined weight fractions. The observed compositional bias may be the result of a combination of all previously identified sources of errors. Nevertheless, the QPA of pyrite concentrate powder ball milled to 3 µm particle size (7 min milling time) produces weight fractions within 2 wt% of those determined using calibrated WD-XRF, with standard deviations <0.3 wt%, confirming the reproducibility of the method and making it suitable at least for comparative analysis.

## 4. Conclusions

The quantitative phase analysis using XRD of pyrite ore concentrate powder extracted from the Thackaringa mine in Australia was problematic due to the presence of large particles, hard impurities and preferred orientation of the albite phase. Milling of the powder to particle sizes around 3 µm yielded reproducible results across preparations and was found to be suitable for the quantitative phase analysis of pyrite concentrate using XRD. Complementary techniques were necessary for the detection of trace elements and minerals. Refined phase fractions using XRD data for all phases are significantly correlated with the mean volume particle size.

Milling resulted in a slight broadening of XRD reflections, without appreciable phase transformation, although substantial broadening of the pyrrhotite phase was observed which may influence the phase quantification after 10 min milling.

The elemental composition obtained from XRD of pyrite concentrate powder milled for 7 min, with a mean volume particle size around 3 µm, was in general agreement with that obtained from WD-XRF. However, we note that some discrepancy remains, perhaps as a result of unaccounted elemental substitutions or amorphous phases.

The study provides a useful methodology for the precise QPA of pyrite ore concentrates using XRD, as long as the analyst is aware of the possible drawbacks of the methods as highlighted in this article. Researchers are urged not to rely on a single preparation or analysis technique for the QPA of pyrite ores and to assess reproducibility and accuracy for each new sample composition with as much knowledge about the sample as possible.

## Supplementary Material

Crystal structure: contains datablock(s) QPAPBMXGreaseSuspendedSamplePrep_publ, QPAPBMXGreaseSuspendedSamplePrep_overall, QPAPBMXGreaseSuspendedSamplePrep_phase_2, QPAPBMXGreaseSuspendedSamplePrep_phase_0, QPAPBMXGreaseSuspendedSamplePrep_phase_3, QPAPBMXGreaseSuspendedSamplePrep_phase_4, QPAPBMXGreaseSuspendedSamplePrep_phase_1, QPAPBMXGreaseSuspendedSamplePrep_pwd_0. DOI: 10.1107/S1600576722009888/vb5042sup1.cif

Crystal structure: contains datablock(s) QPAPBMXHandGrindSamplePrep_publ, QPAPBMXHandGrindSamplePrep_overall, QPAPBMXHandGrindSamplePrep_phase_2, QPAPBMXHandGrindSamplePrep_phase_0, QPAPBMXHandGrindSamplePrep_phase_3, QPAPBMXHandGrindSamplePrep_phase_4, QPAPBMXHandGrindSamplePrep_phase_1, QPAPBMXHandGrindSamplePrep_pwd_0. DOI: 10.1107/S1600576722009888/vb5042sup2.cif

Crystal structure: contains datablock(s) QPABAT3_publ, QPABAT3_overall, QPABAT3_phase_2, QPABAT3_phase_0, QPABAT3_phase_3, QPABAT3_phase_4, QPABAT3_phase_1, QPABAT3_pwd_0. DOI: 10.1107/S1600576722009888/vb5042sup3.cif

Crystal structure: contains datablock(s) QPABAT1ug3_publ, QPABAT1ug3_overall, QPABAT1ug3_phase_2, QPABAT1ug3_phase_0, QPABAT1ug3_phase_3, QPABAT1ug3_phase_1, QPABAT1ug3_phase_4, QPABAT1ug3_pwd_0. DOI: 10.1107/S1600576722009888/vb5042sup4.cif

Crystal structure: contains datablock(s) NEW1minmill_publ, NEW1minmill_overall, NEW1minmill_phase_0, NEW1minmill_phase_2, NEW1minmill_phase_4, NEW1minmill_phase_1, NEW1minmill_phase_3, NEW1minmill_pwd_0. DOI: 10.1107/S1600576722009888/vb5042sup5.cif

Crystal structure: contains datablock(s) NEW2minmill_publ, NEW2minmill_overall, NEW2minmill_phase_0, NEW2minmill_phase_2, NEW2minmill_phase_4, NEW2minmill_phase_1, NEW2minmill_phase_3, NEW2minmill_pwd_0. DOI: 10.1107/S1600576722009888/vb5042sup6.cif

Crystal structure: contains datablock(s) NEW3minmill_publ, NEW3minmill_overall, NEW3minmill_phase_0, NEW3minmill_phase_2, NEW3minmill_phase_4, NEW3minmill_phase_1, NEW3minmill_phase_3, NEW3minmill_pwd_0. DOI: 10.1107/S1600576722009888/vb5042sup7.cif

Crystal structure: contains datablock(s) NEW5minmill_publ, NEW5minmill_overall, NEW5minmill_phase_0, NEW5minmill_phase_2, NEW5minmill_phase_4, NEW5minmill_phase_1, NEW5minmill_phase_3, NEW5minmill_pwd_0. DOI: 10.1107/S1600576722009888/vb5042sup8.cif

Crystal structure: contains datablock(s) NEW10minmill_publ, NEW10minmill_overall, NEW10minmill_phase_0, NEW10minmill_phase_2, NEW10minmill_phase_4, NEW10minmill_phase_1, NEW10minmill_phase_3, NEW10minmill_pwd_0. DOI: 10.1107/S1600576722009888/vb5042sup9.cif

Additional figures and tables. DOI: 10.1107/S1600576722009888/vb5042sup10.pdf

CCDC references: 2223099, 2223100, 2223101, 2223102, 2223103, 2223104, 2223105, 2223106, 2223107, 2223108, 2223109, 2223110, 2223111, 2223112, 2223113, 2223114, 2223115, 2223116, 2223117, 2223118, 2223119, 2223120, 2223121, 2223122, 2223123, 2223124, 2223125, 2223126, 2223127, 2223128, 2223129, 2223130, 2223131, 2223132, 2223133, 2223134, 2223135, 2223136, 2223137, 2223138, 2223139, 2223140, 2223141, 2223142, 2223143

## Figures and Tables

**Table 1:** Crystalline phases identified from XRD data of pyrite powder concentrate ball milled for 7 min

Phase name	Formula	Matching crystal structure reference
Pyrite	FeS_2_	Wu *et al.* (2004 ▸)
Quartz α	SiO_2_	Antao *et al.* (2008 ▸)
Albite, low	NaAlSi_3_O_8_	Armbruster *et al.* (1990 ▸)
Pyrrhotite, 4M	Fe_7_S_8_	Morimoto *et al.* (1975 ▸)
Rutile	TiO_2_	Shintani *et al.* (1975 ▸)

**Table 2:** Mean elemental weight fractions of pyrite concentrate powder ball milled for 7 min determined using semi-quantitative WD-XRF data, and standard deviations from five repeat samples The *K*α peak of oxygen was visible but O was excluded from the analysis due to the lack of accuracy for this element. The presence of trace elements with overlapping energies, such as As *K*α and Pb *L*α, or W *L*β and Au *L*α, was confirmed by ICP-MS (Table S1). Other elements are below the detection/quantification limit of the technique (Loubser & Verryn, 2008 ▸; Kadachi & Al-Eshaikh, 2012 ▸). Elemental fractions were obtained from fundamental parameter analysis.

Element	Wt%	Element	Wt%
S	52.2 (6)	Mg	0.032 (4)
Fe	35.8 (3)	K	0.03 (10)
Si	4.51 (10)	Zr	0.01 (2)
Al	0.95 (2)	W	<0.01
Na	0.74 (4)	As	<0.01
Ti	0.57 (4)	Pb	<0.01
Co	0.45 (2)	Nd	<0.01
Ni	0.058 (8)	Y	<0.01
Ca	0.042 (13)		

**Table 3:** Volume moment mean diameter *D*[4,3] from laser diffraction of ball-milled pyrite concentrate powder samples

Milling time (min)	Mean particle size *D*[4,3] (µm)
0	129 (3)
1	42 (3)
2	10.5 (2)
3	5.62 (14)
5	3.83 (8)
7	2.72 (11)
10	2.19 (11)

**Table 4:** Mean phase fractions obtained from Rietveld analysis using XRD data from five samples of the same pyrite ore concentrate aliquot, each individually milled for 7 min Sample standard deviations of the mean values from the five repeats are indicated. Note the similarity to the estimated standard deviations from Rietveld analysis.

Phase	Pyrite	Albite	Quartz	Pyrrhotite	Rutile
Mean phase fraction (wt%)	82.7 (3)	8.4 (2)	4.94 (12)	3.07 (10)	0.92 (2)

**Table 5:** Comparison of the mean elemental weight fractions obtained using XRD and calibrated WD-XRF weight fractions for five pyrite concentrate samples from the same aliquot, each individually ball milled for 7 min WD-XRF data were empirically calibrated using standard mixtures to correct for matrix effects (Rousseau, 2006 ▸). Sample standard deviations of the mean values from the five repeats are indicated.

	Wt%	
Element	XRD	WD-XRF
S	45.4 (2)	47.2 (2)
Fe	40.3 (2)	42.04 (14)
Si	5.00 (11)	4.03 (10)
