# Supplementary material for: Preparation of pyrite concentrate powder from the Thackaringa mine for quantitative phase analysis using X-ray diffraction
Source: J Appl Crystallogr. 2022 Nov 29;55(Pt 6):1572–82. doi: 10.1107/S1600576722009888 (PMC9721321; doi:10.1107/S1600576722009888)
Supplement: Supplementary file 10 [file j-55-01572-sup10.pdf]

JOURNAL OF APPLIED CRYSTALLOGRAPHY

Volume 55 (2022)

Supporting information for article:

Preparation of pyrite concentrate powder from the Thackaringa mine for quantitative phase analysis using X-ray diffraction

Hamish McDougall, Monica Hibberd, Andrew Tong, Suzanne Neville, Vanessa Peterson and Christophe Didier

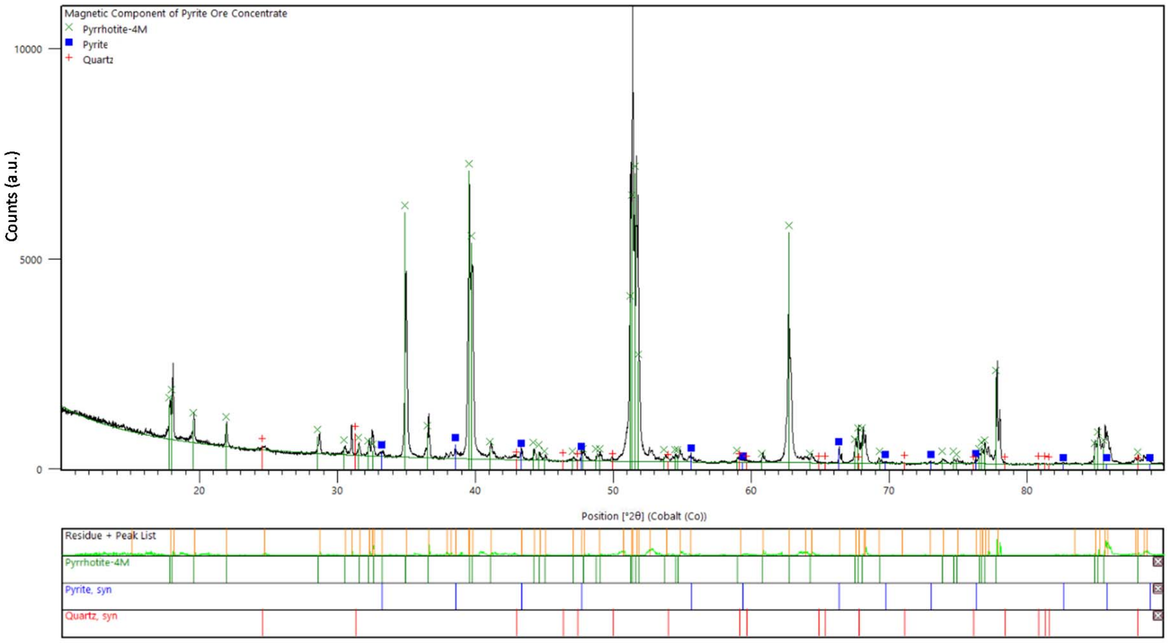

Figure S1 Phase identification using the Highscore software analysis of XRD data of the magnetic component separated from pyrite concentrate powder, hand ground for 15 min prior to measurement. Most reflections are indexed with the 4M polytype of pyrrhotite (Morimoto et al., 1975). A few minor reflections of pyrite and quartz are visible due to imperfect magnetic separation. Data are shown as a black line.

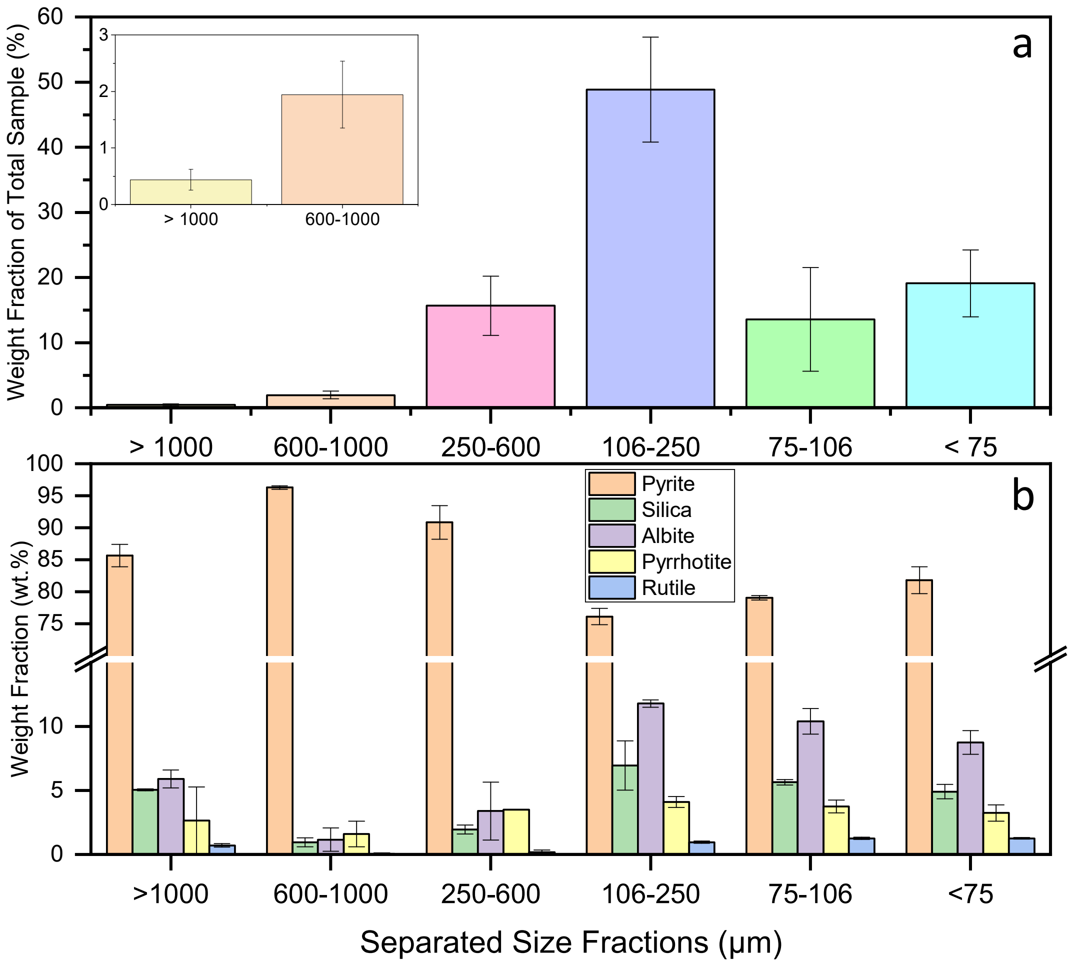

Figure S2 (a) Overall weight distribution of sample recovered from pyrite concentrate across different sized sieves (b) Phase weight fractions within each sieved size fractions determined using XRD of pyrite concentrate powder.

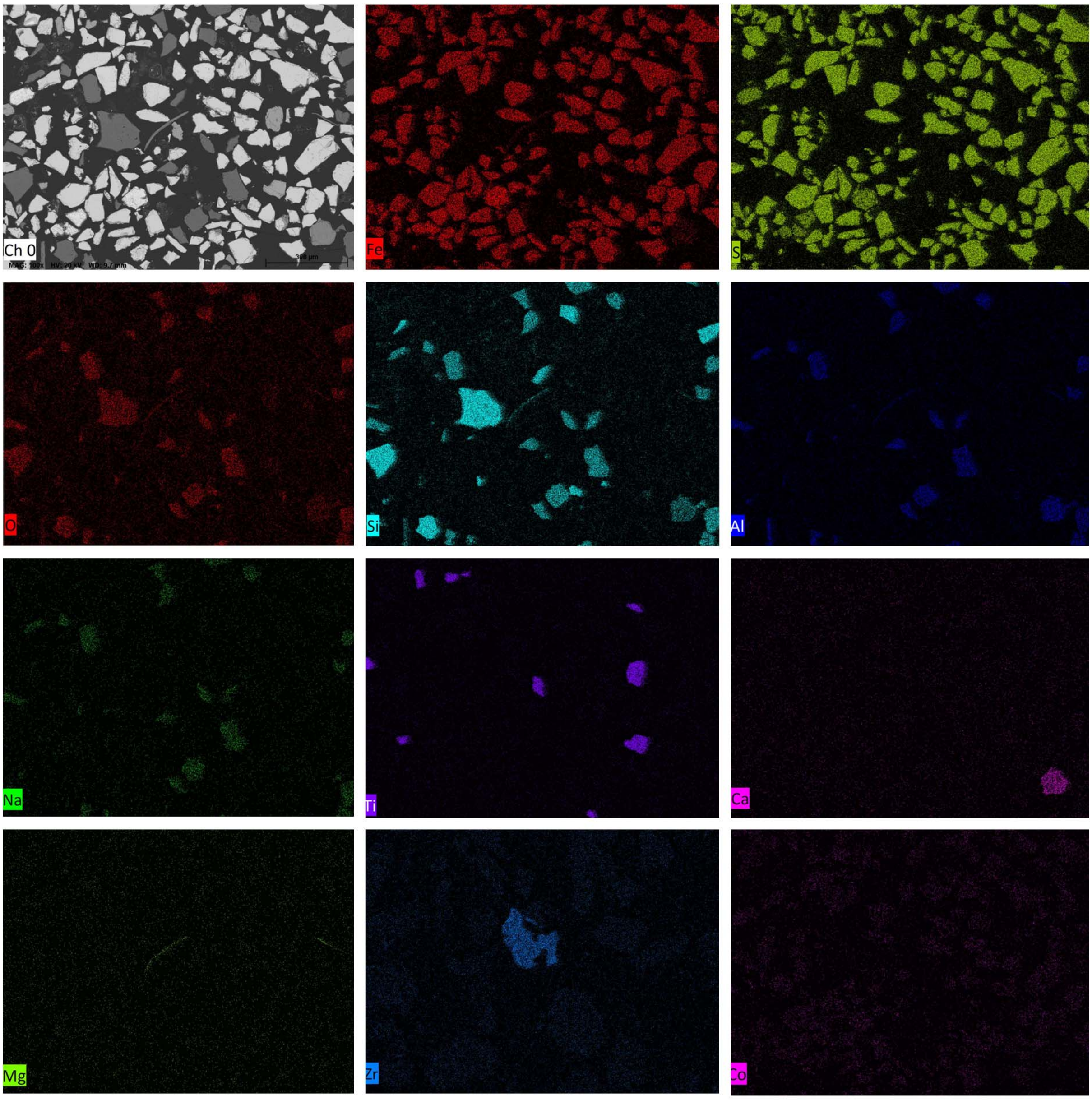

Figure S3 SEM-EDS elemental maps of pyrite concentrate powder sample encased in resin. Zr map is shown for a distinct SEM-EDS image of pyrite concentrate powder sample featuring a single particle with a high concentration of Zr.

Table S1 ICP-MS analysis of elements known to substitute within pyrite crystal lattice, and from which confidence in WD-XRF results could be determined.

Element	Concentration (ppm)
**As**	77.586
**Au**	<MDL*
**Pb**	16.701
**W**	57.37

*MDL – Method detection limit for this method was 1ppm for the elements analysed (the lowest concentration at which an analyte can be detected in the sample with 99% certainty).

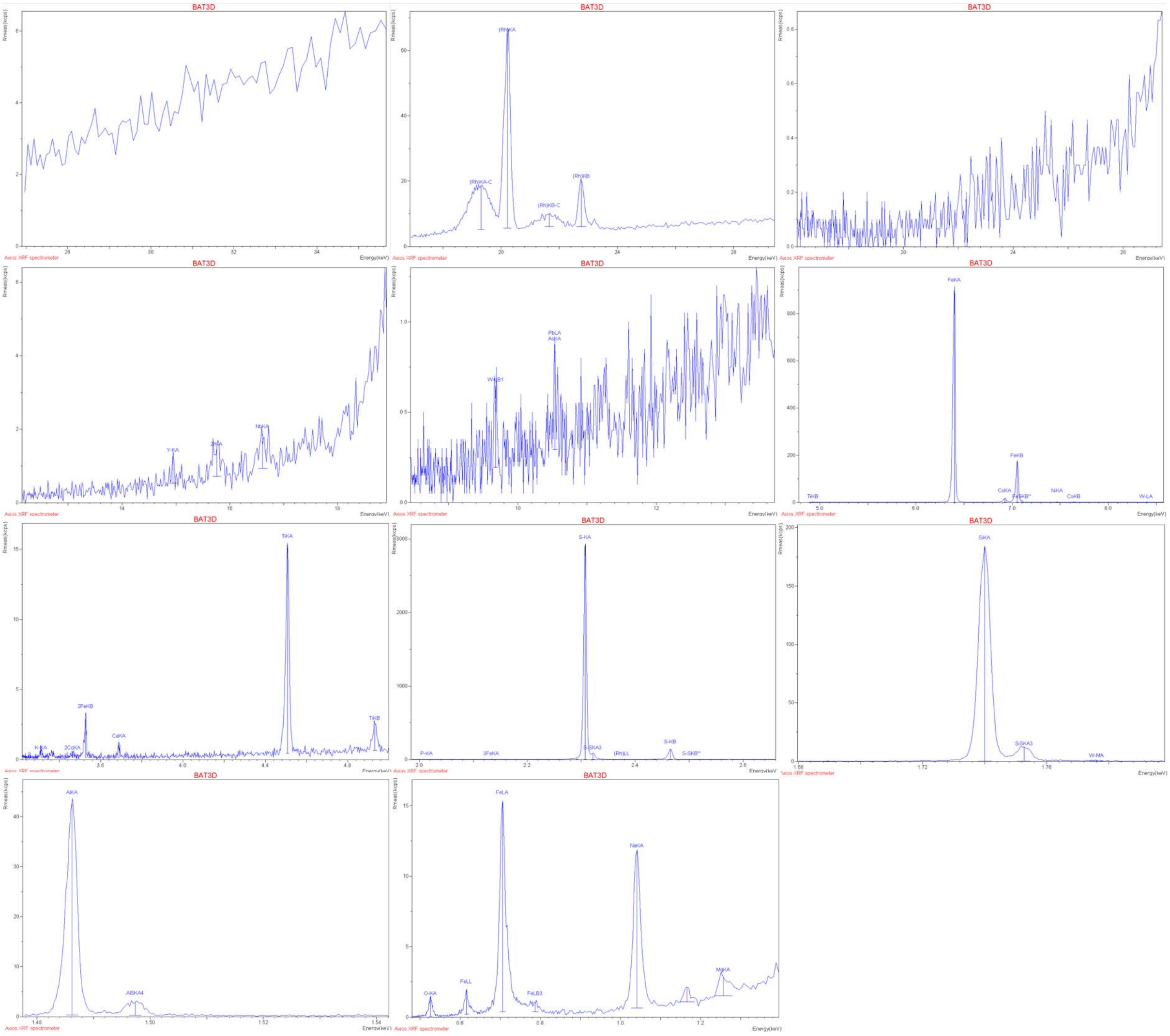

Figure S4 Raw XRF spectra (0-35.3 keV) obtained from a single measurement of pyrite concentrate representative of 5 replicates using the Malvern Panalytical 'Omnian-method' software.

Table S2 Rietveld refinement weighted profile R-factor (R_{wp}), Goodness of Fit (GOF), Bragg R-factors (RF^2), and other refinement parameters using XRD data of pyrite concentrate powder prepared by various methods. Project crystallographic information files from refinement using each technique are linked to this article publication. Standard deviations are estimated from the refinement procedure. GOF is derived from the reduced chi squared from profile refinement as calculated by Toby (2006). * = fixed.

	**Untreated**	**Hand Ground**	**Grease Loaded**	**Ball Milled 7 min**
**R_{wp} (%)**	33.192	15.172	11.428	7.869
**GOF**	8.74	2.51	1.79	1.95
**RF^2 (%)**				
**Pyrite**	22.921	7.544	5.164	3.546
**Albite**	63.254	44.798	39.579	15.033
**Quartz**	42.111	27.236	19.071	6.029
**Pyrrhotite**	38.273	20.760	11.077	11.206
**Rutile**	45.095	19.671	11.859	7.362
**Phase Space Group**				
**Pyrite**	$Pa\bar{3}$			
**Albite**	$C\bar{1}$			
**Quartz**	$P3_221$			
**Pyrrhotite**	$C2/c$			
**Rutile**	$P4_2/mnm$			
**Refined Unit Cell Parameters (Å)**				
**Pyrite**	a = 5.41961(8)	a = 5.4189(2)	a = 5.41927(5)	a = 5.41941(3)
**Albite**	a = 8.317*<br>b = 12.785*<br>c = 7.158*<br>α = 94.26*<br>β = 116.6*<br>γ = 87.71*	a = 8.317*<br>b = 12.785*<br>c = 7.158*<br>α = 94.26*<br>β = 116.6*<br>γ = 87.71*	a = 8.317*<br>b = 12.785*<br>c = 7.158*<br>α = 94.26*<br>β = 116.6*<br>γ = 87.71*	a = 8.317*<br>b = 12.785*<br>c = 7.158*<br>α = 94.26*<br>β = 116.6*<br>γ = 87.71*
**Quartz**	a = 4.9147(6)<br>c = 5.4059(5)	a = 4.9133(3)<br>c = 5.4056(3)	a = 4.9149(5)<br>c = 5.4057(4)	a = 4.914(6)<br>c = 5.4067(5)
**Pyrrhotite 4M**	a = 11.92(2)<br>b = 6.874(4)<br>c = 12.81(3)	a = 11.93(4)<br>b = 6.858(5)<br>c = 12.89(4)	a = 11.937(11)<br>b = 6.863(2)<br>c = 12.811(13)	a = 11.95(3)<br>b = 6.857(4)<br>c = 12.83(3)

	β = 117.20(5)	β = 117.09(7)	β = 117.25(2)	β = 117.25(6)
**Rutile**	a = 4.5835(13)<br>c = 2.9806(12)	a = 4.5959(11)<br>c = 2.9622(11)	a = 4.599(2)<br>c = 2.9616(13)	a = 4.5980(13)<br>c = 2.9619(13)
**Weight Fraction**				
**Pyrite**	0.413(7)	0.752(3)	0.754(3)	0.809(3)
**Albite**	0.320(6)	0.092(2)	0.128(2)	0.085(2)
**Quartz**	0.242(3)	0.0800(11)	0.0550(13)	0.0600(11)
**Pyrrhotite**	0.025(3)	0.072(2)	0.0600(13)	0.0350(11)
**Rutile**	0.001(2)	0.0050(5)	0.0040(4)	0.0110(7)
**Crystallite Size (μm):**				
**Pyrite**	0.36(2)	0.389(6)	0.379(4)	0.181(1)
**Albite**	0.192(12)	0.0920(14)	0.202(13)	0.111(8)
**Quartz**	0.22(6)	0.262(13)	0.24(2)	0.145(6)
**Pyrrhotite 4M**	0.14(5)	0.0221(14)	0.061(4)	0.031(3)
**Rutile**	0.1*	0.1*	0.1*	0.07(1)
**Atomic Coordinates**				
**Pyrite**	Fe(x = y = z)<br>= 0<br>S(x = y = z)<br>= 0.3906(4)	Fe(x = y = z)<br>= 0<br>S(x = y = z)<br>= 0.3887(2)	Fe(x = y = z)<br>= 0<br>S(x = y = z)<br>= 0.38435(13)	Fe(x = y = z)<br>= 0<br>S(x = y = z)<br>= 0.38468(9)
**Isotropic Atomic Displacement Parameters (U_{iso}), Å^2**				
**Pyrite**	Fe = 0.01*<br>S = 0.01*	Fe = 0.0281(1)<br>S = 0.0310(8)	Fe = 0.0115(7)<br>S = 0.0087(6)	Fe = 0.0051(3)<br>S = 0.0058(3)
**March Dollase Ratio**				
**Albite [013]**	0.247(6)	0.424(6)	0.94(1)	0.921(8)

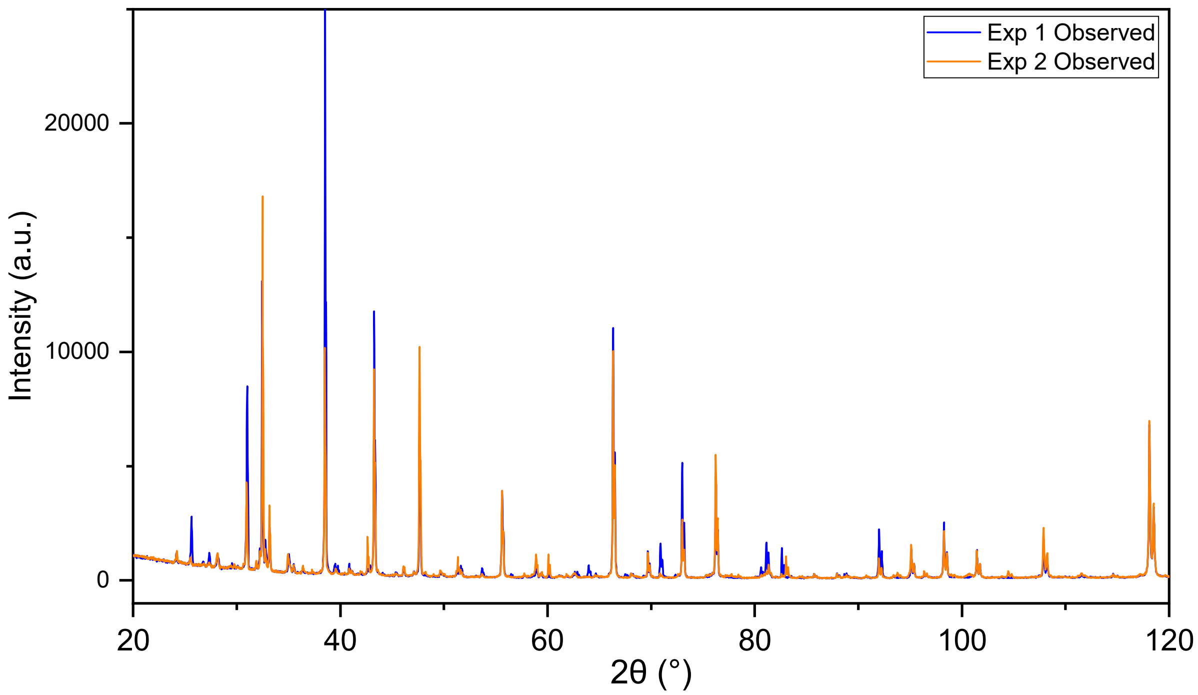

Figure S5 Observed diffraction patterns from XRD data taken from 2 identically treated hand ground pyrite concentrate powder samples demonstrating inconsistency of data. Rietveld refinement of these data was performed, but not shown here for clarity of the profile comparison, with weighted profile R-Factors are R_{wp} = 27.768% and R_{wp} = 24.646% for experiment 1 and 2 respectively.

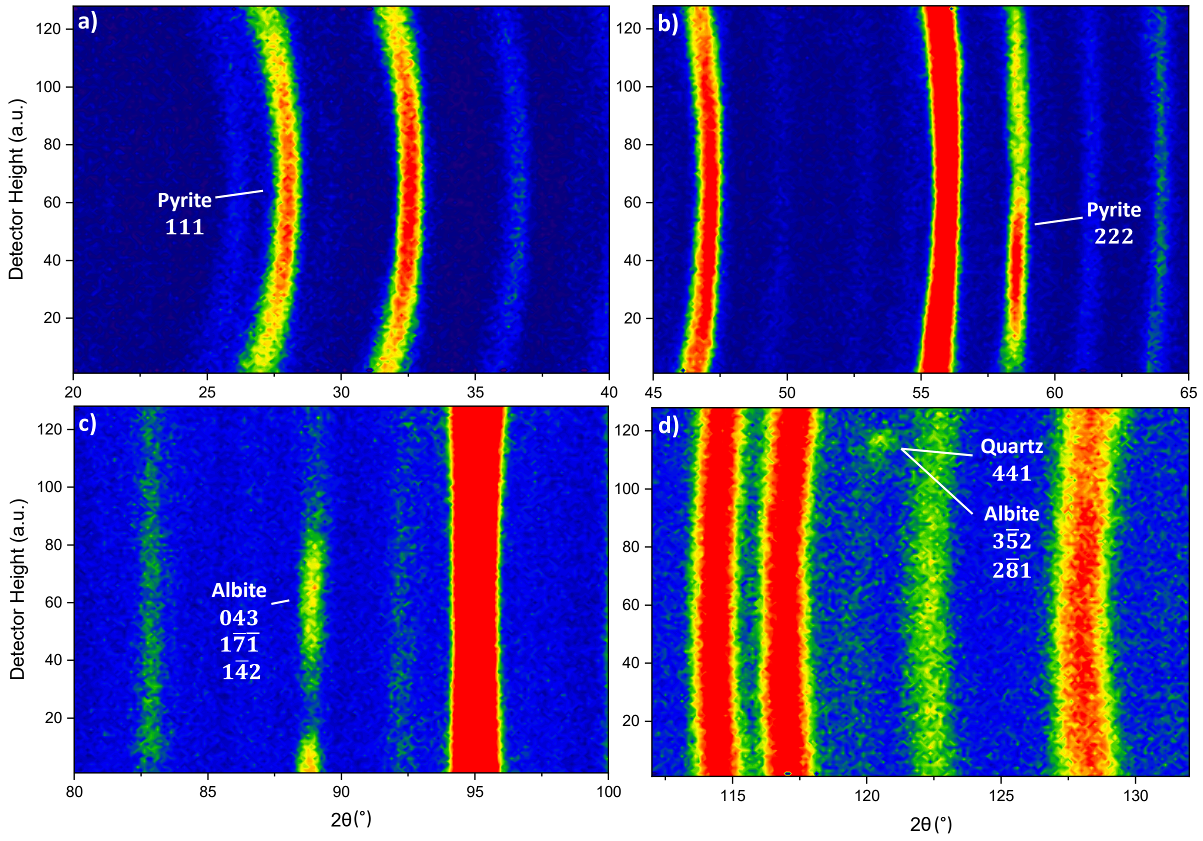

Figure S6 Selected regions of ND data of untreated pyrite concentrate powder collected using an area detector using neutrons a) and b) of 1.5430(1) Å showing preferred orientation for the pyrite reflection 111 at ~ 27 ° and 222 at ~ 58 °, and c) and d) of 2.4144(3) Å showing preferred orientation for the overlapping albite reflections 043, 1$\bar{7}\bar{1}$, 2$\bar{4}$2 at ~ 88.5 °, and for overlapping quartz 441 and albite 3$\bar{5}$2 and 2$\bar{8}$1 reflections at ~ 121 °. Intensity is shown in colour from lowest (blue) to highest (red). ND data of the same sample after a slight rotation of the sample container around the vertical axis resulted in significant changes to the distribution of intensity for these reflections. The spottiness of the Debye Scherrer cones in ND data of the untreated pyrite concentrate sample is consistent with the presence of large crystallites. Neutrons are very penetrating and provide data from a much larger sample volume compared to X-rays, usually resulting in a better statistical representation of all crystallite orientations and a reduction of preferred orientation. Despite the larger investigated volume, the measured Debye-Scherrer cones in ND data of an untreated pyrite concentrate sample arising from albite, pyrite, and quartz, were inhomogeneous.

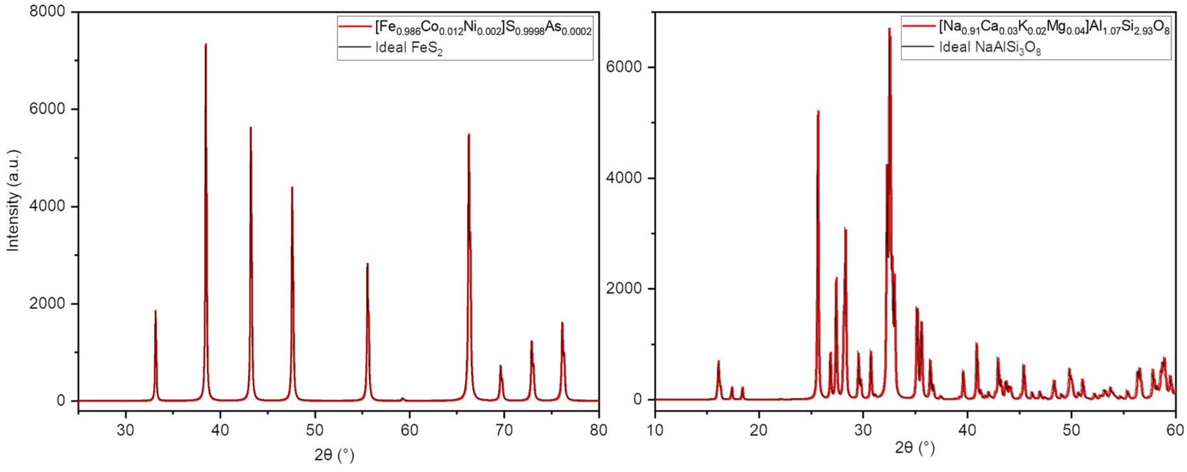

Figure S7 Pattern simulations for pyrite and albite phase for the pure and substituted phases according to multi-elemental WD-XRF. Unit-cell, broadening and atomic parameters were kept constant.

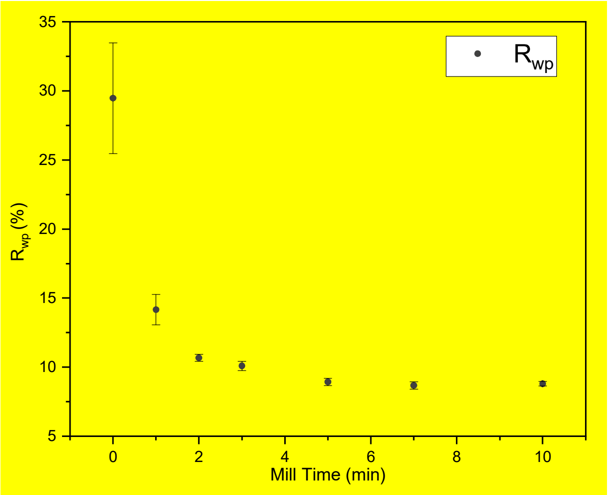

Figure S8 Average weighted profile R-factor (R_{wp}) from Rietveld refinement using XRD data for 5 pyrite concentrate powder samples milled for different milling times. Error bars represent sample standard deviations of the mean values for the five repeats at each milling time.

Table S3 Mean weighted profile residual values (R_{wp}), goodness of fit (GOF), Bragg residual factor (R_F^2) and mean March-Dollase ratio for albite for Rietveld refinements using XRD data of milled pyrite concentrate powder samples milled for different times. Sample standard deviations of the mean values from the five repeats at each milling time are indicated. Representative project crystallographic information files of 1 refinement at each milling time are linked to this article publication. GOF is derived from the reduced chi squared from profile refinement as calculated by Toby (2006).

Mill time (min)	Mean weighted profile R-factor R_{wp} (%)	Goodness of Fit (GOF)	Albite Bragg R-factor R_F^2 (%)	Mean March-Dollase ratio for albite with the unique reflection direction [013], shown in Figure 7.
0	29(4)	7(2.5)	69(7)	0.33(9)
1	14(1)	3.9(8)	31(3)	0.80(6)
2	10.7(3)	2.9(4)	19(2)	0.75(4)
3	10.1(3)	2.57(4)	18(2)	0.80(3)
5	8.9(3)	2.18(6)	15.3(9)	0.87(3)
7	8.7(3)	2.09(7)	15.0(8)	0.94(3)
10	8.8(2)	2.18(14)	14.7(6)	0.95(2)

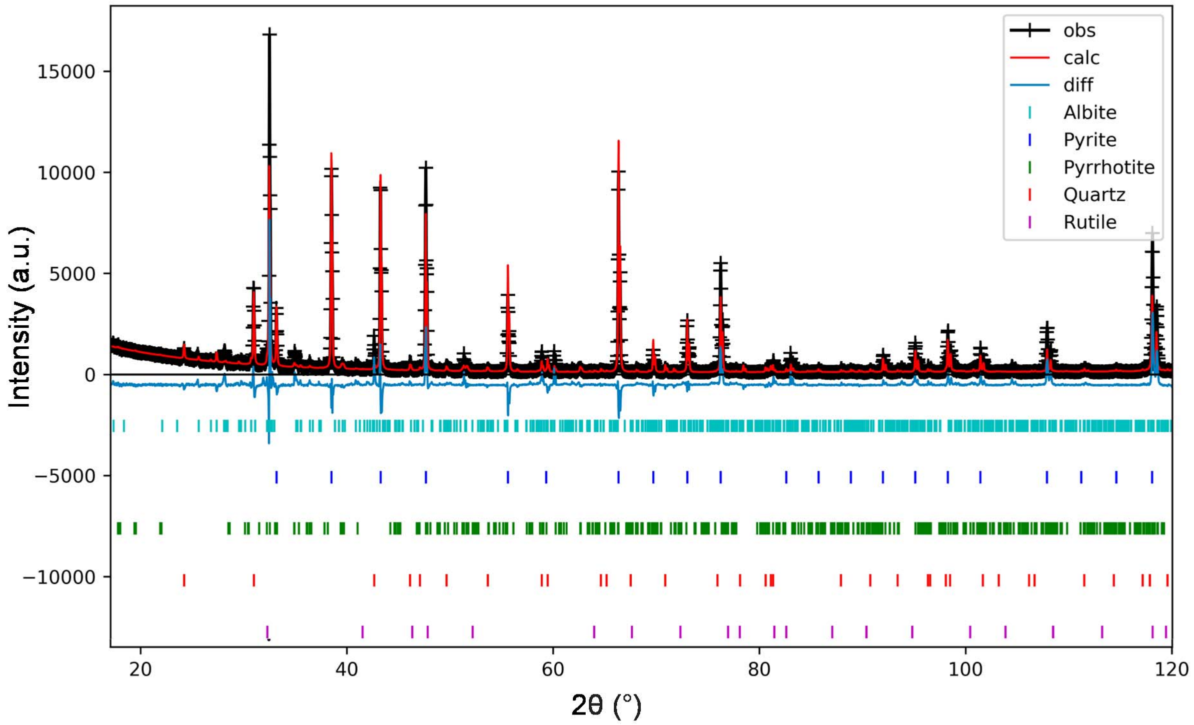

Figure S9 Typical Rietveld refinement profile using XRD data of unmilled pyrite concentrate powder. Difference line is calculated minus observed intensity.

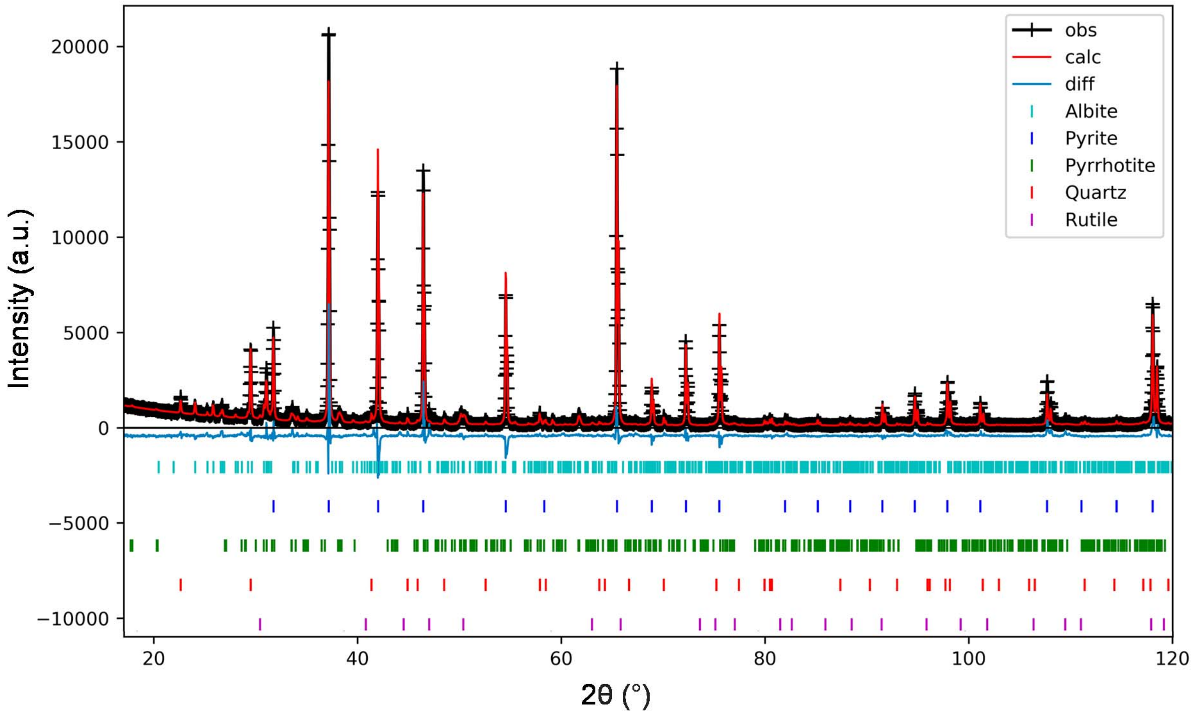

Figure S10 Typical Rietveld refinement profile using XRD data of pyrite concentrate powder milled for 1 min. Difference line is calculated minus observed intensity.

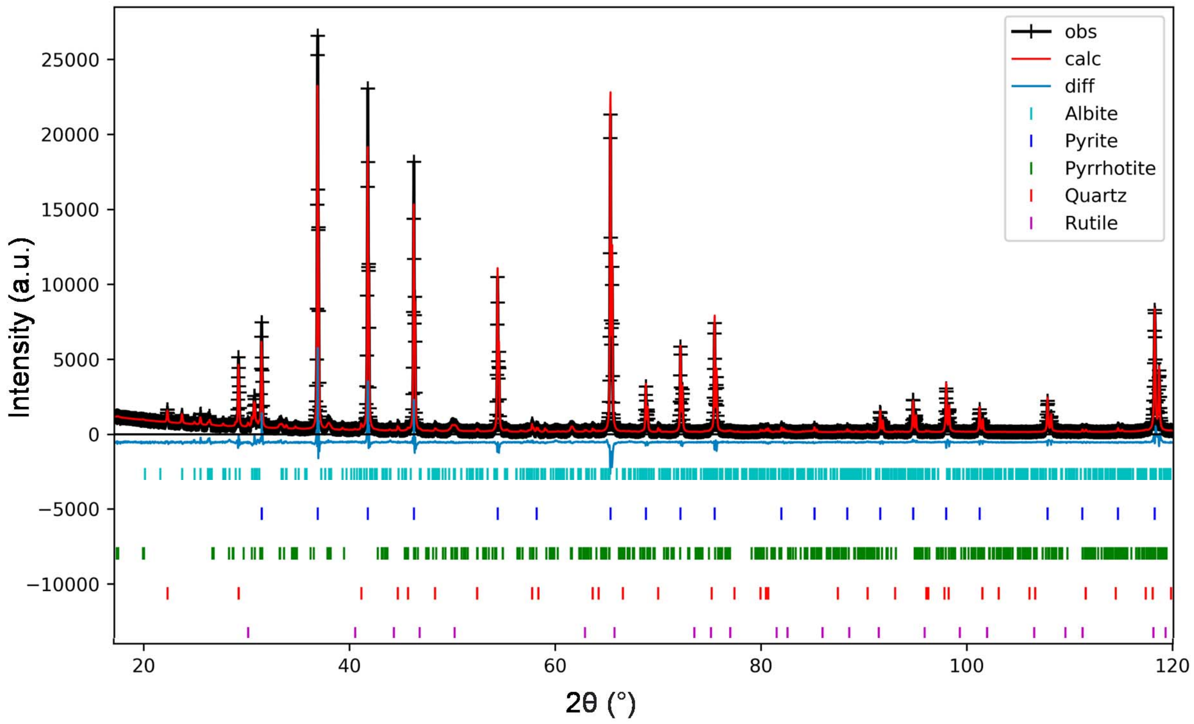

Figure S11 Typical Rietveld refinement profile using XRD data of pyrite concentrate powder milled for 2 min. Difference line is calculated minus observed intensity.

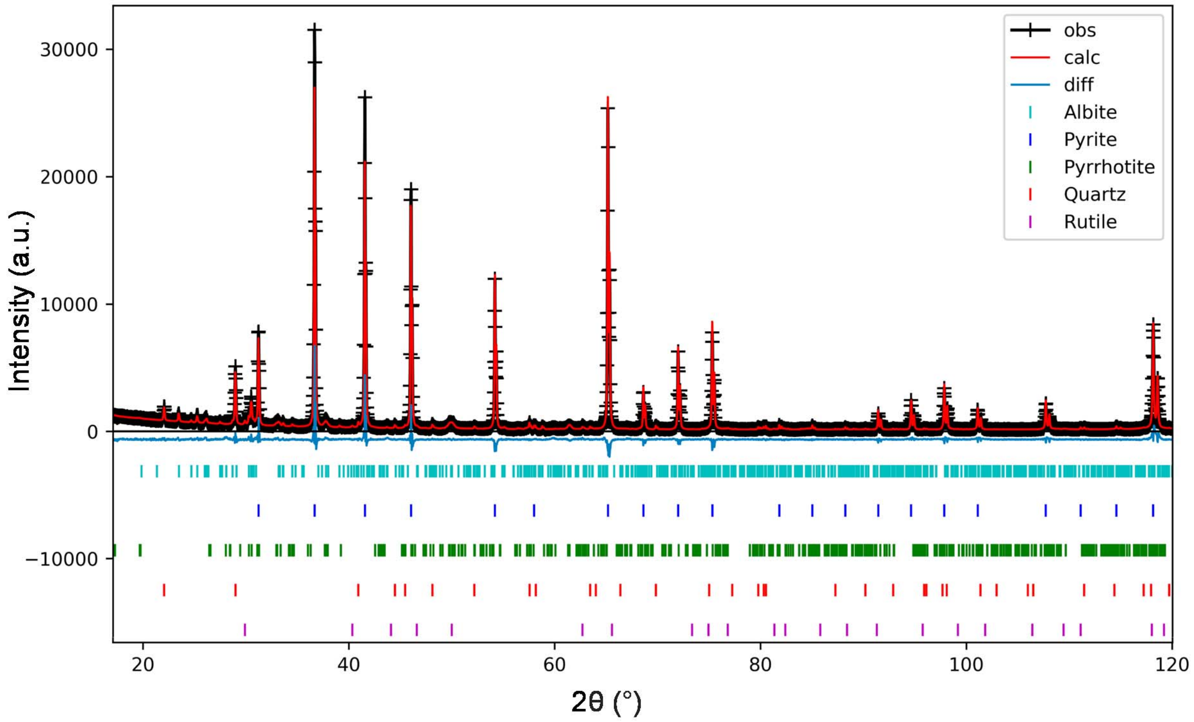

Figure S12 Typical Rietveld refinement profile using XRD data of pyrite concentrate powder milled for 3 min. Difference line is calculated minus observed intensity.

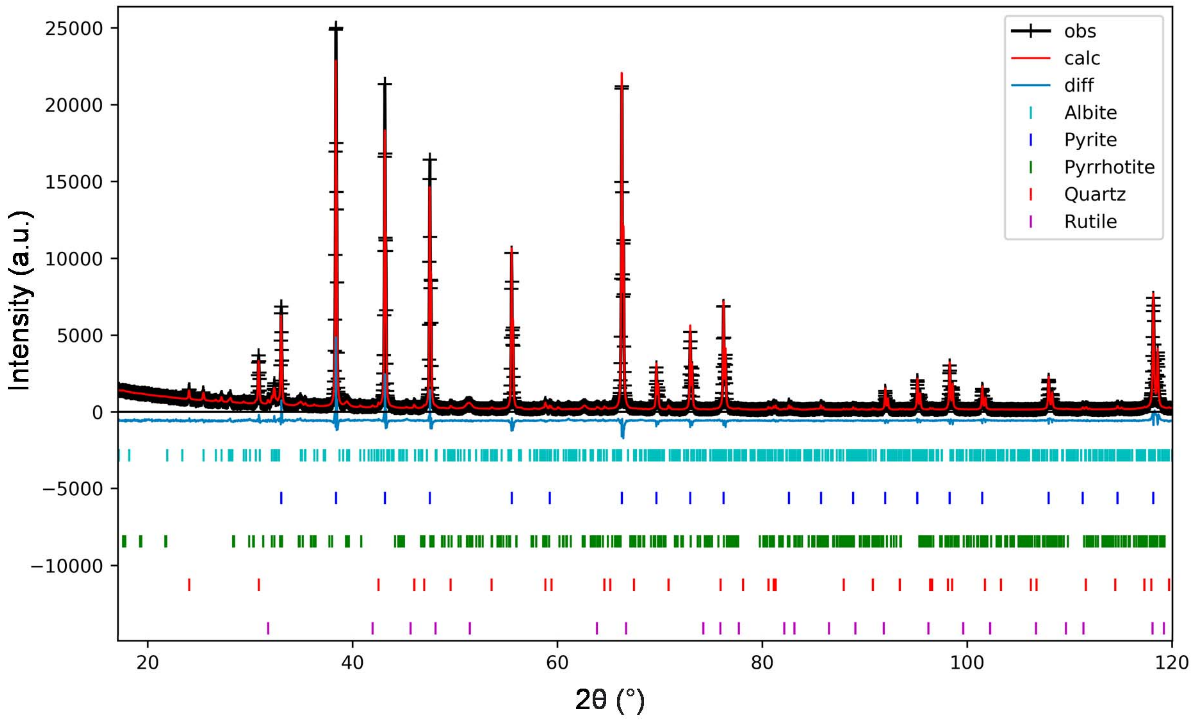

Figure S13 Typical Rietveld refinement profile using XRD data of pyrite concentrate powder milled for 5 min. Difference line is calculated minus observed intensity.

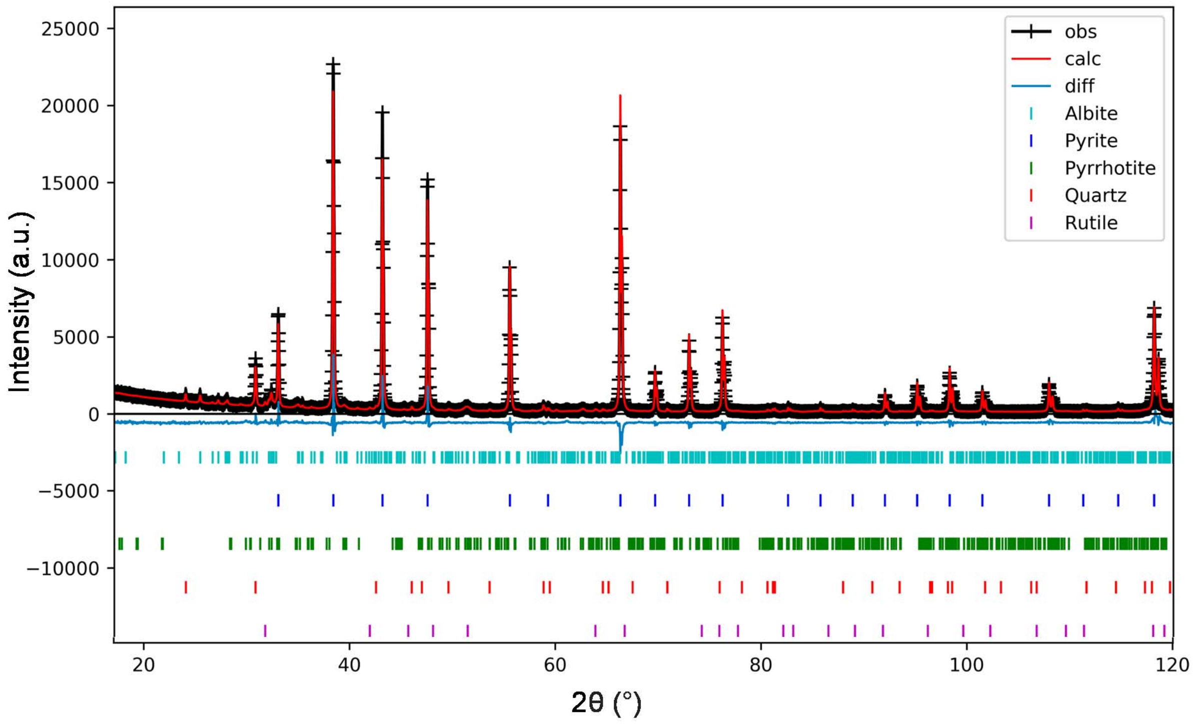

Figure S14 Typical Rietveld refinement profile using XRD data of pyrite concentrate powder milled for 7 min. Difference line is calculated minus observed intensity.

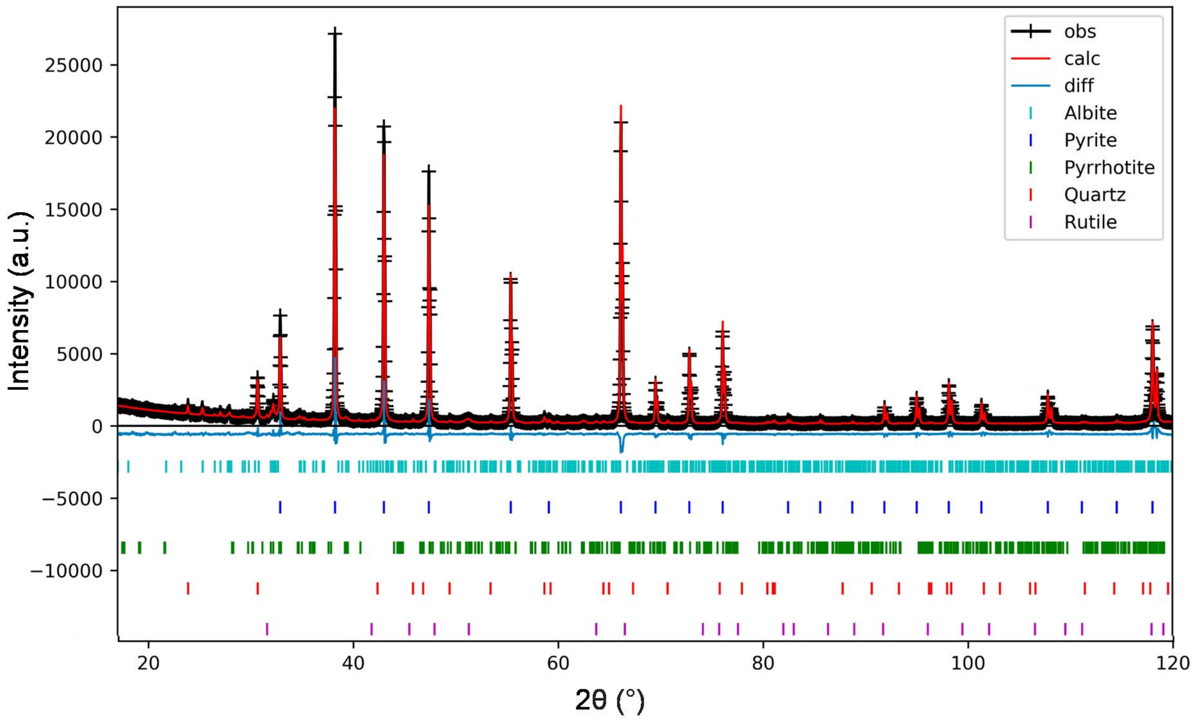

Figure S15 Typical Rietveld refinement profile using XRD data of pyrite concentrate powder milled for 10 min. Difference line is calculated minus observed intensity.

Table S4 Mean unit cell parameters for main crystalline phases from Rietveld refinement using XRD data of 5 pyrite concentrate powder samples prepared with different milling time. Standard deviations were determined from five repeats at each milling time. * = fixed.

	Unit Cell Parameters (Å)		
	Pyrite	Quartz	
Mill Time (min)	a	a	c
0	5.4195(2)	4.8(3)	5.4061(5)
1	5.41921(9)	4.9140(4)	5.4052(9)
2	5.41911(3)	4.9145(2)	5.4047(4)
3	5.41909(3)	4.9145(2)	5.4051(4)
5	5.41918(4)	4.91465(9)	5.4055(3)
7	5.41930(3)	4.9151(2)	5.4054(4)
10	5.41932(2)	4.9153(13)	5.4060(4)
	Pyrrhotite		
Mill Time (min)	a	b	c
0	11.916(9)	6.8(1)	12.810(4)
1	11.902(2)	6.883(2)	12.812(4)
2	11.899(8)	6.886(5)	12.817(4)
3	11.9000(13)	6.886(2)	12.818(2)
5	11.895(3)	6.8897(12)	12.818(4)
7	11.891(4)	6.891(2)	12.818(3)
10	11.893(5)	6.889(4)	12.820(8)
	Albite		
Mill Time (min)	a	b	c
0	8.137*	12.785*	7.1583*
1	8.137*	12.785*	7.1583*
2	8.137*	12.785*	7.1583*
3	8.137*	12.785*	7.1583*
5	8.1418(9)	12.80(2)	7.1580(7)
7	8.1420(12)	12.795(13)	7.1580(10)
10	8.1424(11)	12.7970(2)	7.1590(5)
	Albite (continued)		
Mill Time (min)	α	β	γ
0	94.26*	116.6*	87.71*
1	94.26*	116.6*	87.71*
2	94.26*	116.6*	87.71*
3	94.26*	116.6*	87.71*
5	94.3(2)	116.6(6)	87.78(11)
7	94.27(3)	116.608(5)	87.78(2)
10	94.28(2)	116.622(7)	87.784(10)
	Rutile		
Mill Time (min)	a	c	

0	4.60(8)	2.98(6)
1	4.597(3)	2.963(2)
2	4.5965(12)	2.9611(7)
3	4.5973(9)	2.9611(6)
5	4.5977(9)	2.9614(5)
7	4.5969(11)	2.962(2)
10	4.597(1)	2.9617(13)

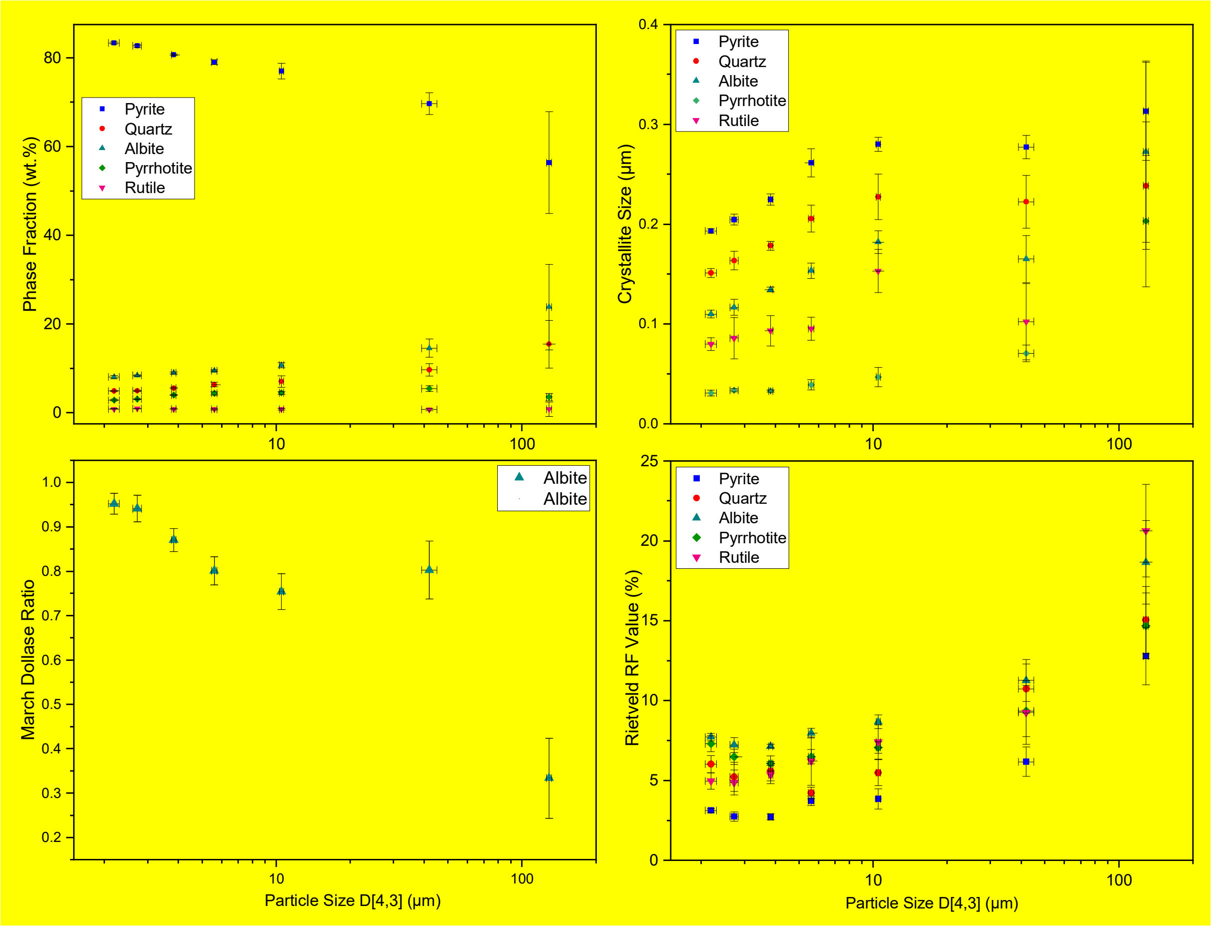

Figure S16 Refined parameters from QPA of pyrite ore concentrates milled between 0-10 min plotted against mean moment volume particle size D4,3 determined from laser diffraction. Vertical error bars represent sample standard deviations of the mean values for the five XRD preparation and analysis repeats at each milling time. Horizontal bars represent sample standard deviations of the mean values for the three laser diffraction preparation and analysis repeats.

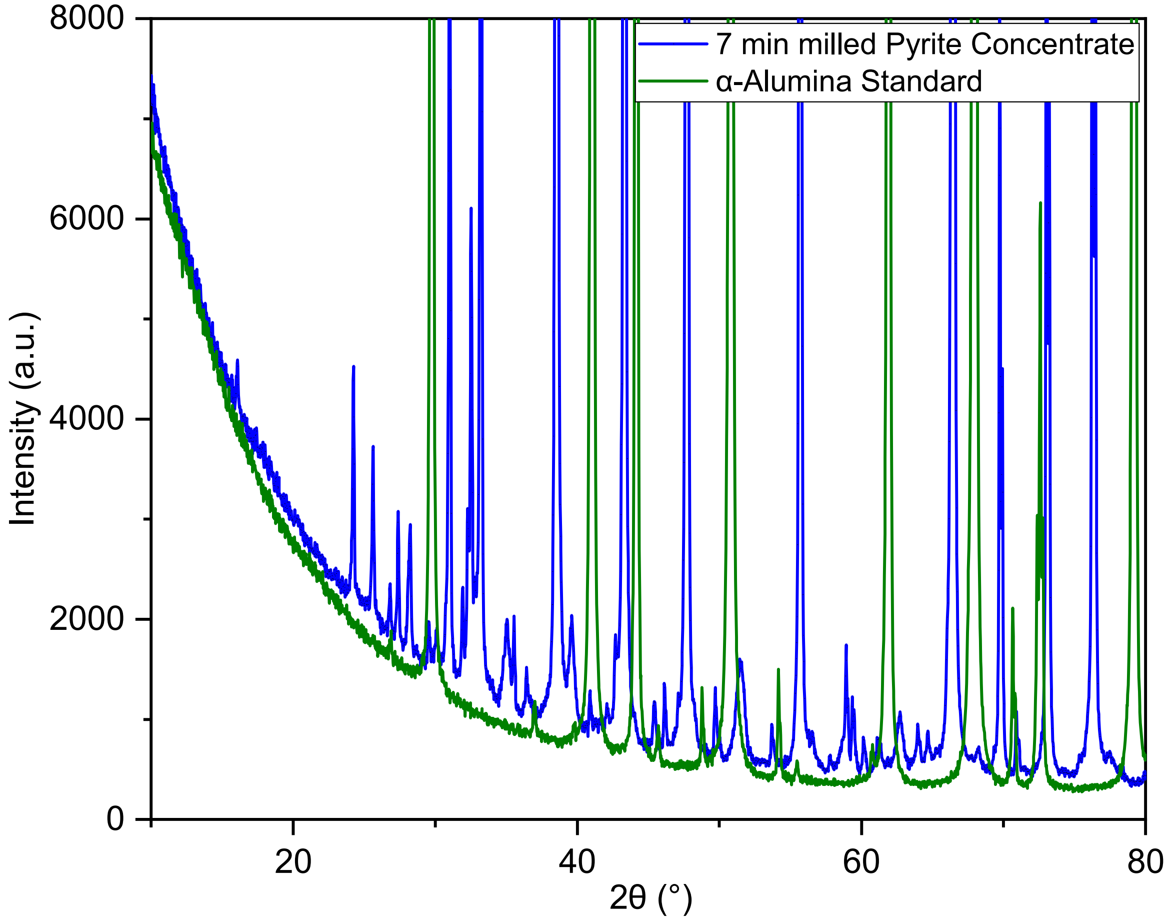

Figure S17 Observed diffraction patterns from XRD data of 7 min milled pyrite concentrate (blue) and crystalline Alumina standard (green) showing absence of broad features indicative of amorphous content.

Table S5 Refined unit-cell, crystallite size, atomic parameters and reliability factors obtained from Rietveld refinement of alpha alumina used for the external standard amorphous content estimation. Errors are estimated standard deviations from the fit.

**a (Å)**	**c (Å)**	**Crystallite size (µm)**	**R_F (%)**	**R_{wp} (%)**
4.75959(6)	12.99285(11)	0.2030(11)	1.88	7.64
**Atom**	**x**	**y**	**z**	**U_{iso} (Å^2)**
Al	0	0	0.35209(4)	0.0056(2)
O	0.3065(2)	0	1/4	0.0080(3)

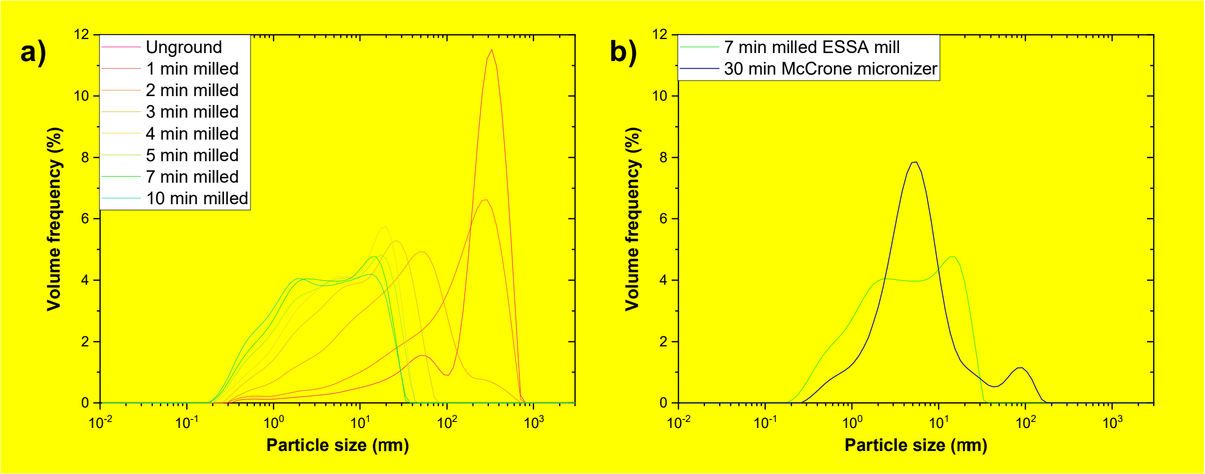

Figure S18 a) Particle size distribution from laser scattering of pyrite concentrate samples milled for different times using the ESSA ball mill. b) Particle size distribution from laser scattering of pyrite concentrate samples milled for 7 min with the ESSA ball mill and for 30 min with the McCrone micronizer. Each measurement was repeated 3 times to confirm observed modes are not the result of sampling.

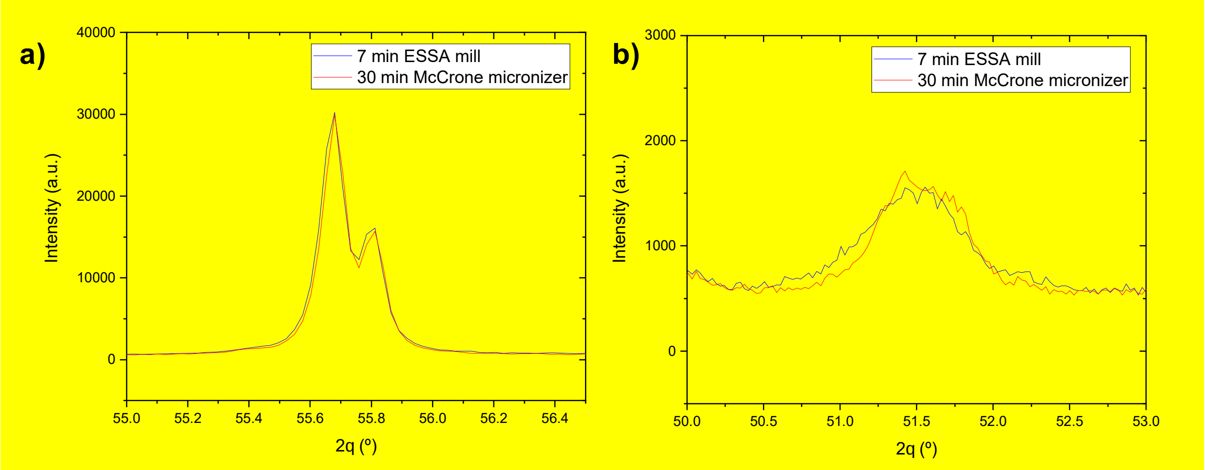

Figure S19 XRD data, shown as a line for clarity, of pyrite concentrate powder milled for 7 min using the ESSA mill and for 30 min using the McCrone micronizer. (a) pyrite overlapped reflections 312 and 321 and (b) pyrrhotite overlapped reflections 402, 223, $22\bar{5}$, $40\bar{6}$ and 313.

Table S6 Refined parameters from Rietveld refinement of pyrite concentrate samples milled for 7 min using the ESSA mill and milled for 30 min using the McCrone mill. Those were prepared from a different aliquot than that used in the remainder of the manuscript, resulting in slightly altered phase ratios due to ore variations.

	**7 min ESSA mill**	**30 min McCrone micronizer**
**R_{wp} (%)**	6.685	6.385
**GOF**	2.65	2.59
	**R_F^2 (%)**	
**Pyrite**	2.731	2.479
**Albite**	7.793	6.843
**Quartz**	16.544	16.625
**Pyrrhotite**	9.220	6.482
**Rutile**	13.035	10.027
	**Refined Unit Cell Parameters (Å)**	
**Pyrite**	a = 5.41877(2)	a = 5.41848(2)
**Albite**	a = 8.143(7)<br>b = 12.792(2)<br>c = 7.158(5)<br>α = 94.261(12)<br>β = 116.615(13)<br>γ = 87.80(2)	a = 8.140(5)<br>b = 12.791(2)<br>c = 7.157(4)<br>α = 94.278(9)<br>β = 116.600(10)<br>γ = 87.78(14)
**Quartz**	a = 4.9147(4)<br>c = 5.4058(4)	a = 4.9136(4)<br>c = 5.4053(4)
**Pyrrhotite 4M**	a = 11.94(3)<br>b = 6.862(4)<br>c = 12.84(3)<br>β = 117.25(5)	a = 11.890(11)<br>b = 6.883(2)<br>c = 12.809(13)<br>β = 117.16(2)
**Rutile**	a = 4.5983(12)<br>c = 2.9593(10)	a = 4.5947(12)<br>c = 2.9594(10)
	**Weight Fraction (%)**	
**Pyrite**	79.9(2)	79.7(2)
**Albite**	8.69(14)	8.57(14)
**Quartz**	6.03(7)	6.48(7)

**Pyrrhotite**	4.33(10)	3.93(8)
**Rutile**	1.08(6)	1.31(6)
**Crystallite Size (μm):**		
**Pyrite**	0.301(2)	0.358(3)
**Albite**	0.148(10)	0.187(12)
**Quartz**	0.209(9)	0.181(7)
**Pyrrhotite 4M**	0.0290(2)	0.0511(3)
**Rutile**	0.100(12)	0.0686(7)
**Atomic Coordinates**		
**Pyrite**	Fe (x = y = z) 0<br>S (x = y = z) 0.38474(8)	Fe (x = y = z) 0<br>S (x = y = z) 0.38476(7)
**Isotropic Atomic Displacement Parameters (U_{iso}), Å^2**		
**Pyrite**	Fe = 0.0012(2)<br>S = 0.0002(2)	Fe = 0.0023(2)<br>S = 0.0011(2)
**March Dollase Ratio**		
**Albite [013]**	0.886(11)	0.872(9)
